# PyLoGreen: Design and implementation of a low-cost agricultural data acquisition and monitoring system using Raspberry Pi, LoRa, and nRF24L01 in the High-Andean Tundra

**DOI:** 10.1016/j.ohx.2026.e00766

**Published:** 2026-04-04

**Authors:** Renzo Victor Carpio Hallasi, Yusef Mamani Arqque, Franco Alessandro Arenas Mamani, Alejandro Enrique Contreras Corzo, Pablo Lizardo Pari Pinto, Erasmo Sulla Espinoza

**Affiliations:** a School of Electronic Engineering, National University of San Agustin, Arequipa, Peru; b Department of Electronic Engineering, National University of San Agustin, Arequipa, Peru

**Keywords:** LoRa, Raspberry Pi, MicroPython, Instrumentation, Monitoring, Low-cost

## Abstract

This article presents PyLoGreen, an open-source hardware platform that enables continuous monitoring of air temperature, relative humidity, soil moisture, soil pH, and internal and external light levels inside greenhouses located in the High-Andean tundra of Juliaca (3824 m a.s.l.). The system is designed as a cost-accessible and reproducible scientific platform for regions where commercial greenhouse monitoring solutions are either unavailable or economically inaccessible for small-scale farmers. A hybrid architecture, combining long-range LoRa links (up to 7.5 km validated in dedicated line-of-sight tests with 100% packet delivery, and 2.53 km operational deployment) between a Raspberry Pi 4B Main Base and a remote greenhouse with a local nRF24L01 sensor network based on Raspberry Pi Pico nodes, is implemented to ensure reliable operation under harsh climatic conditions and limited connectivity. A one-month deployment in a real greenhouse demonstrates stable data acquisition from all nodes and robust LoRa communication, validating PyLoGreen as a practical tool for generating high-resolution environmental datasets that can support agronomic and environmental research in high-altitude systems.

## Graphical abstract

**Figure:**
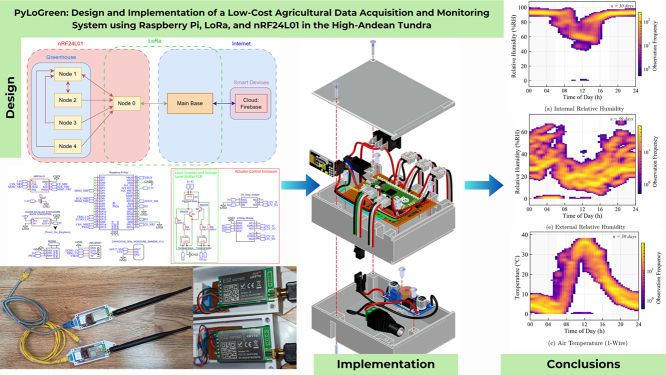

**Table:** 

**Specifications table**	
**Hardware name**	*PyLoGreen*

**Subject area**	•*General*

**Hardware type**	•*Field measurements and sensors* •*Electrical engineering and computer science*

**Closest commercial analog**	*Libelium Smart Agriculture Xtreme*

**Open source license**	*GPL*

**Cost of hardware**	*$367*

**Source file repository**	https://doi.org/10.17605/OSF.IO/APHCV

**OSHWA certification UID**	*PE000003*

## 1 Hardware in context

Global food demand is steadily increasing, especially in urban areas that rely heavily on imported products. Industry 4.0 technologies have significantly improved agricultural productivity in developed countries (for example, in China and the United States), allowing a continuous supply of food even under extreme climatic conditions [1].

Commercial solutions offer advanced monitoring capabilities and robust network integrations. For instance, the Libelium Smart Agriculture Xtreme platform [2] represents the closest commercial analog to our system, employing a distributed wireless sensor network to monitor environmental and soil parameters. However, platforms like Libelium, alongside large-scale systems from companies such as John Deere [3], are often prohibitively expensive, rely on proprietary closed-source architectures, and are designed for large-scale industrial agriculture. This makes their adoption by small producers difficult.

In Peru, the so-called Puna, or high-Andean tundra, is located at 3824 m.a.s.l. and extends mainly across the Altiplano plateau [4]. In regions such as Puno and Juliaca, the harsh climate, characterized by low humidity, strong winds, and nighttime frosts, limits agriculture, which is predominantly small-scale and carried out with limited resources for advanced technologies [5]. Therefore, **PyLoGreen** proposes a scalable and cost-effective system based on microcontrollers such as the Raspberry Pi and LoRa modules, capable of measuring key environmental parameters for agricultural purposes.

**Fig. 1:**
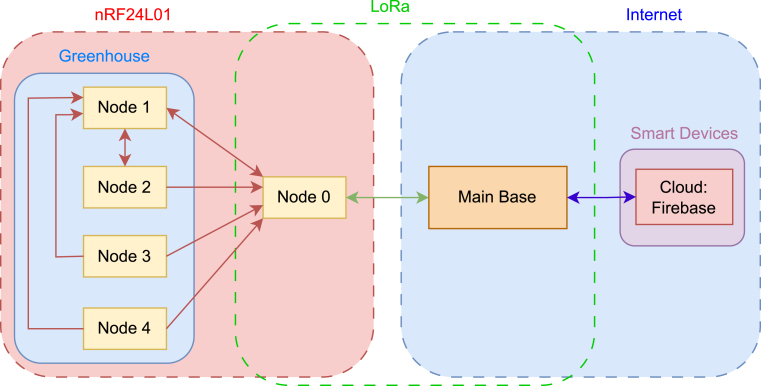
PyLoGreen: Main block diagram.

The Ministry of Agrarian Development and Irrigation (MIDAGRI), through AGRO RURAL, announced in 2024 the implementation of 2560 greenhouses in high-Andean zones, with the goal of guaranteeing food security and reducing malnutrition in vulnerable populations [6].

All these systems require sensors to monitor the health of plants, trees, and crops. Parameters such as humidity and temperature are directly linked to irrigation efficiency, and several applications already integrate LoRa technology in smart irrigation systems [7]. The equipment must also operate reliably under harsh outdoor conditions, enduring temperature variations, humidity, and insects [8].

Greenhouses provide suitable conditions for the proper development of sprouts and allow for higher production per surface area compared to open fields. Furthermore, the yield per unit area is 4 to 5 times higher [9]. Thermodynamically, the structure functions as a passive solar collector: short-wave solar radiation penetrates the transparent cover and is absorbed by the internal surfaces, re-radiating as long-wave thermal energy that is trapped within the enclosure. This process creates a stable microclimate with elevated temperature and humidity relative to the external environment, enabling the cultivation of temperate crops despite the harsh ambient conditions of the high-Andean plateau.

Regarding software, the choice of versatile languages such as Python and MicroPython constitutes a notable difference compared to commercial solutions that typically use C++. This facilitates code maintenance and comprehension for users with basic technical knowledge. However, MicroPython-based algorithms generally show the lowest performance, which is expected since it is an interpreted language and introduces significant computational overhead. Nevertheless, as a high-level language, it simplifies code writing and editing, making development faster and more accessible. While this advantage is less relevant in embedded systems, where efficiency and manual optimization are often required, MicroPython is still highly suitable for general-purpose applications, teaching environments, and student projects [10].

Finally, our solution **PyLoGreen** is complemented with 3D-printed parts for protection, printed circuit boards, and is based on the block diagram shown in Fig. 1, an installation guide to facilitate replication, and field tests conducted, laying the foundations for future improvements.

**Engineering Challenge:** From an engineering standpoint, the main challenge addressed by PyLoGreen is the design of an autonomous monitoring and control system that can operate reliably in remote high-altitude greenhouses, characterized by large daily temperature swings (−10 to $+30{\;}^{\circ }$C), high solar radiation (UV index >11), and limited access to grid power and communication infrastructure. In this environment, conventional greenhouse controllers and Wi-Fi-based data loggers are prone to communication dropouts and premature hardware degradation. PyLoGreen addresses this challenge by combining a long-range LoRa link between the Main Base location and the greenhouse with a low-power nRF24L01 sensor network inside the structure, by distributing the control logic among Raspberry Pi Pico nodes and a Raspberry Pi 4 Main Base, and by using PETG enclosures and custom PCBs to protect the electronics from humidity and temperature fluctuations.

## 2 Hardware description

**PyLoGreen** is a customizable and low-cost solution for agricultural monitoring, developed with open-source hardware. The architecture is specifically tailored to the constraints of high-Andean greenhouses, where the structure may be located several kilometers away from the Main Base location and internet connectivity is intermittent or unavailable. The Main Base of the system consists of a Raspberry Pi 4B equipped with a Quad Core 64-bit ARM Cortex-A72 CPU at 1.5 GHz, 4 GB of LPDDR4 RAM, an SD card slot, and 802.11 b/g/n/ac wireless connectivity [11]. These features make it a suitable option as a server, since it manages cloud communication, coordinates the sensor network, and allows scalability through the incorporation of new distributed acquisition nodes.

The Main Base communicates with the greenhouse nodes through a long-range LoRa transceiver (E32-900T30D), configured to operate at 915 MHz within its 868–930 MHz range, with a reach of up to 8 km [12]. Because the operation within Peru requires adherence to the industrial, scientific, and medical (ISM) radio bands allocated by the National Frequency Allocation Plan (PNAF) [13], this long-range link allows the Main Base to be placed inside the house of the farmer while maintaining communication with the remote greenhouse, overcoming the limitations of Wi-Fi-based systems in this terrain. The module requires a voltage of 3.3–5.2 V and presents an approximate consumption of 650 mA in transmission, 25 mA in reception, and 5 μA in standby mode [12].

Inside the greenhouse, the sensor nodes are based on the Raspberry Pi Pico, a low-cost development board employing the RP2040 microcontroller [14]. It features a dual-core ARM Cortex-M0+ processor running up to 133 MHz, 2 MB of external Flash memory, and 264 kB of internal SRAM. Its 40-pin DIP-style format exposes 26 multifunction GPIOs at 3.3 V, three of which can be used as inputs for its 12-bit ADC. Furthermore, it includes standard peripherals such as 2 UART, 2 I^2^C, 2 SPI, and 16 PWM channels [14]. These nodes are interconnected via a short-range local network implemented with nRF24L01 transceivers, which operate in the 2.46 GHz ISM band, are powered at 1.9–3.6 V, and are configured through a 4-pin SPI interface [15].

Each node integrates a modular sensor suite tailored to crop requirements. In the full deployment, Node 4 is used for differential environmental monitoring by acquiring both internal and external conditions; its external probes (temperature/humidity and light) are routed through the greenhouse wall while keeping the sensitive electronics protected inside the structure (see the deployment description in Section 5.2). Environmental monitoring relies on the digital temperature and humidity sensor (DHT22), which operates from −40 °C to 80 °C on a 3.3–6 V supply [16], and capacitive soil moisture sensors featuring analog output and corrosion-resistant materials [17]. For substrate analysis, the system employs an industrial analog transmitter (RS-PH-∗-TR-1) measuring pH 3-9; it is powered at 12 V and provides dual opposing 0–5 V outputs (with the negative terminal grounded) [18]. The entire system is powered by a 12 V main supply regulated by step-down DC-DC converters (XL4005), which deliver a stable 5 V bus at up to 5 A [19].

The main design features are:

1. **Low cost:** Use of affordable and readily available components.
2. **Easy installation:** 3D-printed protective enclosures that simplify assembly.
3. **Modularity:** Easy replacement of sensors or addition of extra nodes.
4. **Suitable for remote areas:** Prepared for harsh climatic conditions.

This work focuses on the technical architecture and system validation. Agronomic data analysis is beyond its scope and will be addressed in future studies. The data flow and node interaction are detailed in Section 5.

For the node enclosures, PETG (Polyethylene Terephthalate Glycol) was selected due to its good mechanical toughness, with an Izod impact resistance above 4.5 kJ/m^2^, and its intrinsic resistance to the high humidity conditions of the greenhouse. [20].

A digital light sensor module (GY-302, based on the BH1750FVI) from ROHM, communicates via I^2^C, offers 16-bit resolution, and is capable of measuring from 1 to 65,535 lux, operating with a 5 V DC power supply [21].

The JSN-SR04T-3.0 ultrasonic sensor measures non-contact distances in a range of 21–600 cm with an accuracy of ±1 cm [22]. It operates at 3.0–5.5 V DC, with a maximum consumption of 8 mA. It emits pulses at 40 kHz within a 75° angle and supports a temperature range from −20 °C to 70 °C [22].

**PyLoGreen** offers advantages for research applications:

• **Environmental Sciences:** Enables distributed and long-term monitoring of microclimates in remote ecosystems, collecting detailed data on temperature, humidity, and soil conditions for the study of climate change and ecological processes.
• **Agronomy and Crop Sciences:** Facilitates the validation of irrigation strategies, fertilizer analysis through pH and soil moisture, as well as the evaluation of new crop varieties in controlled greenhouses. Its low cost favors replicated experimental designs.
• **IoT and Communications Engineering:** Its hybrid architecture with LoRa and nRF24L01 provides a real-world testbed for developing communication protocols, energy management algorithms, and routing strategies in IoT networks.
• **Low-Cost Scientific Instrumentation:** Serves as an open hardware and software model to create sensor networks adaptable to diverse applications, from water quality monitoring to tracking remote habitats.

## 3 Design files

The necessary files (3D models, Python/MicroPython code, electronic schematics, and some datasheets), summarized in Table 1, are available in the OSF repository: https://doi.org/10.17605/OSF.IO/APHCV.

**Table 1:** List of main design files. Additional files are available in the repository.

Designator	File name	File type	License	Repository/URL
3D01	Bottom_Cover_Node_0	STL	GPL	OSF link
3D02	Bottom_Cover_Nodes_1_2_3_4	STL	GPL	OSF link
3D03	Case_Node_0	STL	GPL	OSF link
3D04	Case_Node_1	STL	GPL	OSF link
3D05	Case_Node_2	STL	GPL	OSF link
3D06	Case_Node_3	STL	GPL	OSF link
3D07	Case_Node_4	STL	GPL	OSF link
3D08	Upper_cover_All_Nodes	STL	GPL	OSF link
3D09	Case_Rele	STL	GPL	OSF link
3D10	Case_LuxSensor	STL	GPL	OSF link
3D11	Case_LoRa	STL	GPL	OSF link
3D12	Cover_for_Case_LoRa	STL	GPL	OSF link
3D13	LoRa_antenna_mount	STL	GPL	OSF link
3D14	Cover_for_DHT22	STL	GPL	OSF link
3D15	Support_RJ45_jack	STL	GPL	OSF link
3D16	Case_JSN-SR04T	STL	GPL	OSF link
3D17	Cover_for_Case_JSN-SR04T	STL	GPL	OSF link
3D18	Cover_for_JSN-SR04T	STL	GPL	OSF link
PY01	main_base	PY	GPL	OSF link
PY02	main_node_0	PY	GPL	OSF link
PY03	main_node_1	PY	GPL	OSF link
PY04	main_node_2	PY	GPL	OSF link
PY05	main_node_3	PY	GPL	OSF link
PY06	main_node_4	PY	GPL	OSF link
PY07	nrf24l01reg	PY	GPL	OSF link
PY08	dht	PY	GPL	OSF link
PY09	LoRa_set_to_915_Mhz	PY	GPL	OSF link
PY10	Lora_Long_Range_TX	PY	GPL	OSF link
PY11	Lora_Long_Range_Rx	PY	GPL	OSF link
JS01	PCB_LoRa	JSON	GPL	OSF link
JS02	PCB_Node_0	JSON	GPL	OSF link
JS03	PCB_Node_1	JSON	GPL	OSF link
JS04	PCB_Node_2	JSON	GPL	OSF link
JS05	PCB_Node_3	JSON	GPL	OSF link
JS06	PCB_Node_4	JSON	GPL	OSF link
JS07	PCB_Relay	JSON	GPL	OSF link
FL01	PCB_LoRa_CopperLayer	PDF	GPL	OSF link
FL02	PCB_LoRa_Silkscreen	PDF	GPL	OSF link
FL03	PCB_Node_0_CopperLayer	PDF	GPL	OSF link
FL04	PCB_Node_0_Silkscreen	PDF	GPL	OSF link
FL05	PCB_Node_1_CopperLayer	PDF	GPL	OSF link
FL06	PCB_Node_1_Silkscreen	PDF	GPL	OSF link
FL07	PCB_Node_2_CopperLayer	PDF	GPL	OSF link
FL08	PCB_Node_2_Silkscreen	PDF	GPL	OSF link
FL09	PCB_Node_3_CopperLayer	PDF	GPL	OSF link
FL10	PCB_Node_3_Silkscreen	PDF	GPL	OSF link
FL11	PCB_Node_4_CopperLayer	PDF	GPL	OSF link
FL12	PCB_Node_4_Silkscreen	PDF	GPL	OSF link
FL13	PCB_Relay_CopperLayer	PDF	GPL	OSF link
FL14	PCB_Relay_Silkscreen	PDF	GPL	OSF link

## 4 Bill of materials summary

A comprehensive list of the components, hardware modules, and raw materials required to build the PyLoGreen system is summarized in Table 2. Detailed specifications, unit costs, and suggested suppliers are included to facilitate replication.

**Table 2:** Bill of materials.

Designator	Component	Number	Cost per unit - currency	Total cost - currency	Source of materials	Material type
RP01	Raspberry Pi 4B, 4 GB	1	$72.17	$72.17	The Pi Box	Electronic
RP02	Raspberry Pi Pico	5	$6.08	$30.40	The Pi Box	Electronic
SN01	DHT22	4	$0.99	$3.96	AliExpress	Electronic
SN02	Capacitive Soil Moisture Sensor V1.2	4	$0.84	$3.36	AliExpress	Electronic
SN03	RS-PH-∗-TR-1 Soil pH Transmitter Analog type	1	$36.70	$36.70	Alibaba	Electronic
SN04	BH1750 Light Intensity Sensor Module	1	$1.38	$1.38	AliExpress	Electronic
SN05	JSN-SR04T Waterproof Ultrasonic Module	1	$4.25	$4.25	AliExpress	Electronic
AC01	MG995 Servo Motor	2	$4.35	$8.70	AliExpress	Electronic
AC02	1/2-inch 12 V Solenoid Valve	1	$4.70	$4.70	AliExpress	Electronic
AC03	12 V 120×120×25 mm Cooling Fans	3	$4.96	$14.88	AliExpress	Electronic
AC04	2-Channel DC 5 V Low-Level Relay Module	1	$2.34	$2.34	AliExpress	Electronic
AC05	1-Channel 5 V Low-Level Relay Module	1	$0.83	$0.83	AliExpress	Electronic
WC01	Transceiver LoRa 868/915MHz E32-900T30D V8	2	$9.63	$19.26	AliExpress	Electronic
WC02	nRF24L01	5	$1.32	$6.60	AliExpress	Electronic
WC03	3000 mm SMA-J to SMA-K Extension Cable (Male to Female)	1	$2.58	$2.58	AliExpress	Electronic
WC04	5000 mm SMA-J to SMA-K Extension Cable (Male to Female)	1	$3.02	$3.02	AliExpress	Electronic
WC05	868MHz/915MHz LoRa 2PCS Antenna 5DBi SMA Male	1	$5.10	$5.10	AliExpress	Electronic
PC01	5Pcs 5.5×2.1 mm Male DC Power Plug	1	$2.08	$2.08	AliExpress	Electronic
PC02	5Pcs 5.5×2.1 mm Female DC Power Jack Adapter	1	$2.07	$2.07	AliExpress	Electronic
PC03	Voltage converter DC-DC Step-Down 5 A XL4005	5	$0.60	$3.00	AliExpress	Electronic
PC04	1Set 150 mm, 2-pin SM JST male/female connectors	6	$0.28	$1.68	AliExpress	Electronic
PC05	5Pcs 15 × 21 mm 2-Position Rocker Switch	1	$1.68	$1.68	AliExpress	Electronic
PC06	5Pcs Male Micro USB Connector	1	$1.85	$1.85	AliExpress	Electronic
PC07	20000 mm UL2468 2-Pin Electric Cable, 22 AWG	1	$5.32	$5.32	AliExpress	Electronic
MC01	5Pcs FR4 PCB 70 × 100 mm Single-Sided Copper-Clad Board	1	$3.34	$3.34	AliExpress	Electronic
MC02	100 μF 16 V capacitor	5	$0.06	$0.30	electromania	Electronic
MC03	10Pcs 1 × 40 Female Header	1	$1.99	$1.99	AliExpress	Electronic
MC04	10Pcs 1 × 40 Male Header	1	$1.67	$1.67	AliExpress	Electronic
MC05	1 kΩ Trimmer Potentiometer	1	$1.32	$1.32	AliExpress	Electronic
MC06	1/4 W 1.8 kΩ resistor	1	$0.01	$0.01	Local electronics store, Lima, Peru	Electronic
MC07	1/4 W 470 Ω resistor	2	$0.01	$0.02	Local electronics store, Lima, Peru	Electronic
MC08	2N2222 NPN transistor	2	$0.19	$0.38	AliExpress	Electronic
MC09	2-pin terminal block	3	$0.59	$1.77	AliExpress	Electronic
MC10	2 × 4 Pin Female Header	5	$0.26	$1.30	AliExpress	Electronic
MC11	1/4 W 1 kΩ resistor	2	$0.01	$0.02	Local electronics store, Lima, Peru	Electronic
IC01	1Set JST-XH 3-Pin Connector	14	$0.40	$5.60	AliExpress	Electronic
IC02	1Set JST-XH 4-Pin Connector	3	$0.60	$1.80	AliExpress	Electronic
IC03	Dixon RJ-45 Cat.5e Punchdown Jack	4	$1.84	$7.36	Dixon	Electronic
IC04	Black Shielded 4-Core Control Cable, 24 AWG 1000 mm	50	$1.00	$50.00	Alibaba	Electronic
IC05	Ethernet Patch Cable (Cat 5e, 1 m)	2	$5.00	$10.00	Local electronics store, Lima, Peru	Electronic
GM01	Phillips Mixed Pan Head Self-Tapping Screws	32	$0.03	$0.96	TORNISA(PE)	Metal
GM02	M3 brass hexagonal spacer, 6 mm body, includes M3 with 6 mm screw and nut	24	$0.36	$8.64	AliExpress	Metal
GM03	M3 with 6 mm Stainless Steel Screw, DIN7985 GB818	7	$0.29	$2.03	AliExpress	Metal
GM04	1 kg White PETG, 1.75 mm	1	$15.80	$15.80	KREAR3D	Plastic
GM05	Ferric Chloride(III)	1	$2.50	$2.50	mastertronic	Chemical
GM06	Transparent Acrylic 2×112×112 mm	1	$2.70	$2.70	BuscalPeru	Acrylic
GM07	PVC JUNCTION BOX 20×20×8 cm LH/IP65	1	$5.86	$5.86	ACSA	PVC
GM08	PVC JUNCTION BOX 15×15×7 cm LH/IP65	1	$3.07	$3.07	ACSA	PVC

*Note:* Approximate total cost: $367 (includes accessories such as cables, power supplies, and shipping).

## 5 Build instructions

### 5.1 System architecture and data flow

**Fig. 2:**
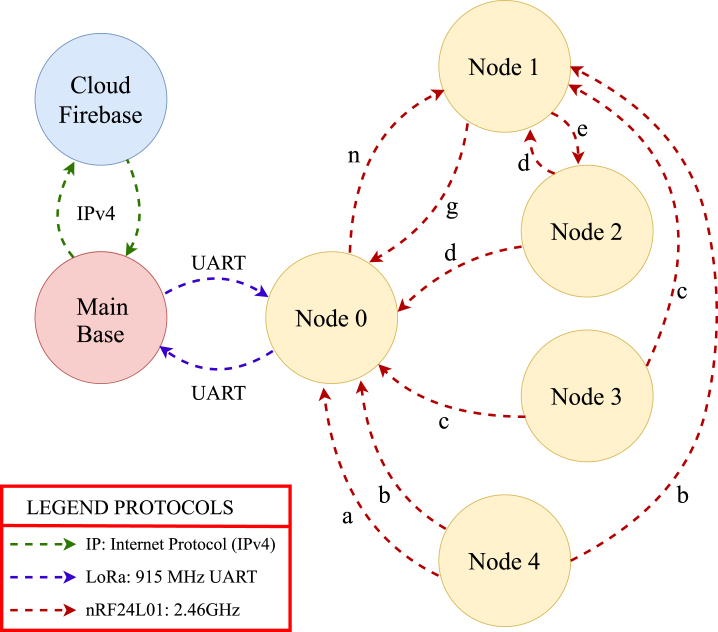
Operational flow and architecture diagram of PyLoGreen.

The system architecture is organized into three logical tiers: the Main Base, the Bridge (Node 0), and the Distributed Sensor Network (Nodes 1-4). This hierarchical structure minimizes power consumption by keeping complex processing and cloud synchronization logic at the Main Base, allowing the distributed sensor nodes to remain lightweight and autonomous (see Table 2). The operational logic follows the state machine model illustrated in Fig. 2. The control cycle originates at the Main Base and propagates through a direct P2P LoRa link (915 MHz) to Node 0. This topology ensures deterministic latency and independence from external gateway infrastructure. The command is then disseminated via the low-latency nRF24L01 network (2.46 GHz) to the sensor nodes. This hybrid topology allows the Main Base to maintain control over a remote greenhouse situated kilometers away, overcoming the range limitations of standard Wi-Fi without sacrificing the data density of the local sensor grid.

**Fig. 3:**
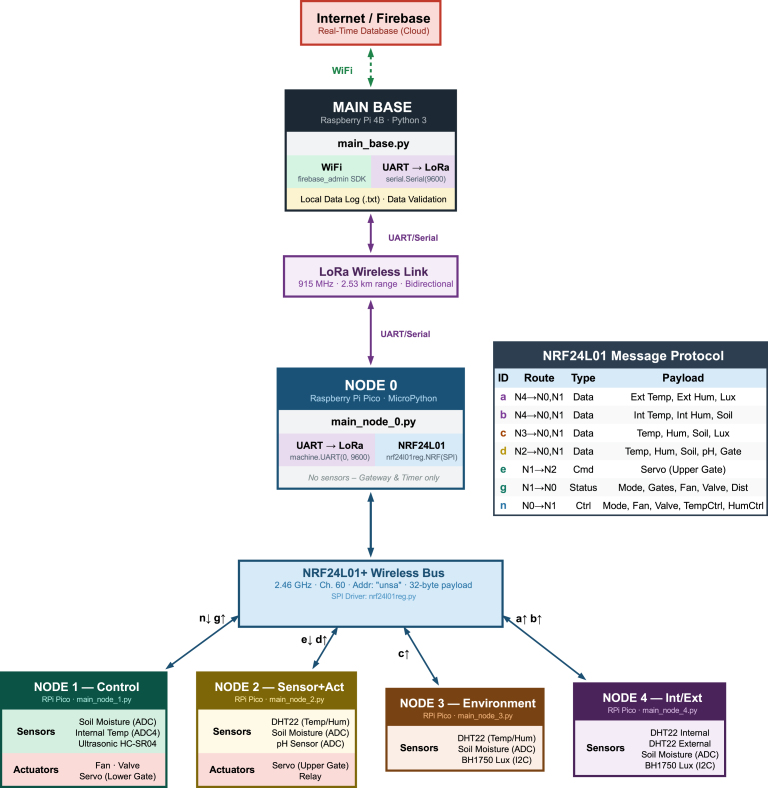
**System architecture and communication data flow.** The diagram details the message routing between nodes, specific data payloads (ID a-n), and the dual-logging strategy used to ensure data integrity in the high-Andean environment.

To detail the specific signal pathways, Fig. 3 maps the system interfaces, identifying the specific communication protocols ($I^{2}C$, 1-Wire, UART) and the distinct packet identifiers (a-n) used for data routing. The data flow is designed to be deterministic and collision-free, operating in a sequential acquisition cycle. The process begins when the Main Base retrieves user-defined parameters from the Firebase cloud, including operation modes, setpoints, and actuator states. These parameters are encoded into a single integer control vector (variable cs) and transmitted via UART to Node 0. This control signal acts as the synchronization trigger for the entire network. Upon receiving this trigger, Node 0 transitions to a polling state to collect data from the internal mesh.

To optimize the 32-byte payload limit of the nRF24L01 transceivers, data is serialized using a prefix-based protocol. The distributed nodes are functionally divided into monitoring and control units. The monitoring function is executed by Nodes 3 and 4, which focus purely on data acquisition. Node 4 utilizes a dual-probe configuration to capture differential metrics by splitting readings into internal and external packets, while Node 3 monitors the microclimate deep within the crop canopy.

Conversely, the control function is handled by Nodes 1 and 2, which combine sensing with electromechanical actuation. Node 1 serves as the primary irrigation controller, managing the water tank levels and solenoid valves, whereas Node 2 governs the ventilation logic. Although these nodes execute local safety routines, they rely on the master command packet from Node 0 to synchronize their actuator states with the central control strategy.

Once the distributed data is collected, Node 0 aggregates the payloads into a 24-value floating-point array and transmits the frame back to the Main Base via LoRa. To address the intermittent internet connectivity typical of high-Andean regions, the Main Base implements a dual-logging strategy. Data is invariably written to a local backup file before any cloud upload attempt is made, ensuring zero data loss even during extended connectivity blackouts. Detailed code is available in Table 7

### 5.2 Physical deployment and topology

**Fig. 4:**
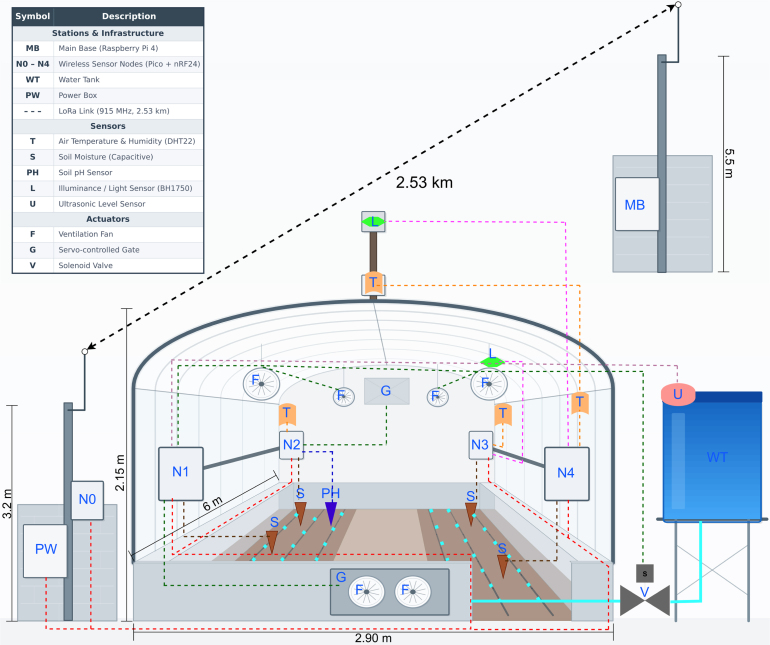
**System deployment and spatial configuration.** The diagram illustrates the physical arrangement of nodes within the high-Andean greenhouse structure.

As illustrated in Fig. 4, the physical deployment translates the logical architecture defined in Figs. 3 and 2 into a real-world setup. Crucially, this spatial configuration was strictly constrained by the geometry of the pre-existing infrastructure. Consequently, the node distribution was leveraged to validate the robustness of the nRF24L01 mesh communication within a non-idealized structural environment, prioritizing the evaluation of electronic link stability and data transmission reliability despite the physical obstacles of the crop and structure.

The nodes were positioned to optimize cabling routes and actuator proximity. Node 1 is located at the front-left corner to manage the water supply and intake ventilation, effectively reducing the necessary cabling length to the main heavy-load actuators. Across the aisle, at the front-right corner near the access door, Node 4 is mounted on the structural frame. This location is specifically chosen to facilitate the routing of its external wired probes (temperature/humidity and light probes) through the greenhouse wall, enabling the comparison of internal versus external conditions.

Deeper inside the structure, Node 2 is situated in the rear-left corner, directly adjacent to the hot air exhaust vent which it actively controls. Finally, Node 3 is placed in the rear-right corner to monitor soil moisture and pH levels at the furthest point from the entrance. To ensure operational durability, all external cabling connecting these distributed points is routed through protective corrugated conduits, safeguarding the system against mechanical wear and the high UV radiation typical of the Altiplano environment.

### 5.3 General description of electronic schematics

**Fig. 5:**
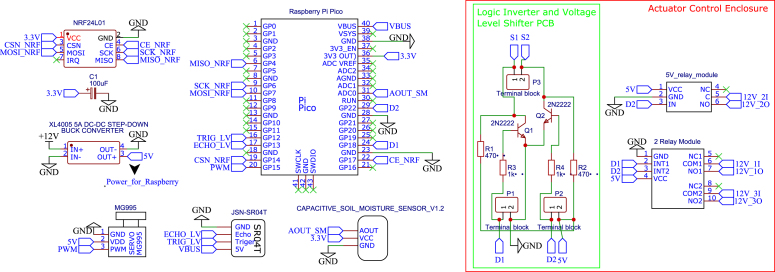
Schematic diagram of Node 1.

The electronic architecture is exemplified by Node 1 (Fig. 5), which demonstrates the standard integration of the Raspberry Pi Pico (RP02) with the sensor array, communication modules, and power management units. In this diagram, the red boundary demarcates the actuator control enclosure, while the green area highlights the Logic Inverter and Voltage Level Shifter PCB. The theoretical basis for the 2N2222 transistor biasing (MC08) used in this stage is detailed in Appendix A, providing the necessary design context before the assembly phase.

**Fig. 6:**
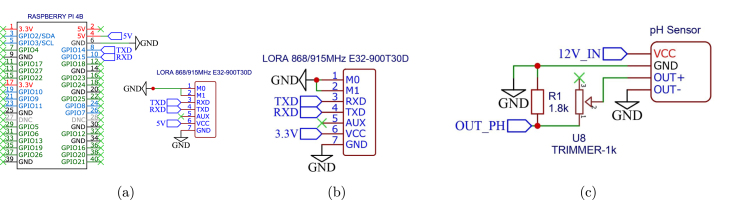
Schematic diagram details: (a) Main Base Diagram, (b) LoRa interface for Node 0, (c) Voltage Divider of Node 2.

Complementing the main node architecture, Fig. 6 details specific sub-circuits crucial for system connectivity and signal conditioning. Fig. 6(a) presents the Main Base schematic, while Fig. 6(b) illustrates the LoRa module interface for Node 0.

Of particular importance is the voltage divider circuit for Node 2 (Fig. 6(c)), designed to interface the pH sensor. The sensor generates a linear analog output ($V_{\text{OUT+}}$) ranging from 0 to 5 V (corresponding to 3–9 pH). To ensure compatibility with the 3.3 V ADC of the microcontroller, the circuit conditions this signal to a safe range ($V_{\text{OUT\_PH}}$ ≤ 3.3 V). The complete mathematical derivation and implementation details are provided in Appendix B.

For the complete schematic diagrams of all remaining nodes, refer to Appendix C.

### 5.4 3D printing and material selection

**Fig. 7:**
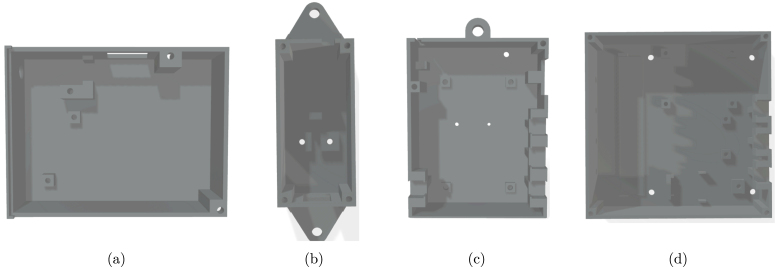
3D modeled components: (a) Bottom cover for Node 0, (b) LoRa module enclosure, (c) Main enclosure for Node 4, (d) Relay control box.

For the fabrication of the structural components, white PETG filament (GM04) was selected. This material choice is critical for the high-Andean context: PETG offers superior thermal resistance and durability compared to PLA, while the white color minimizes solar heat absorption, passively protecting the internal electronic components from overheating (see Fig. 7).

The STL files corresponding to each component are listed in Section 3. To ensure reproducibility, a detailed step-by-step fabrication guide—including specific slicer settings (temperature, speed, infill), the recommended printing sequence, and the detailed list of parts per sensor—is provided in Appendix F.

### 5.5 Printed circuit board design

**Fig. 8:**
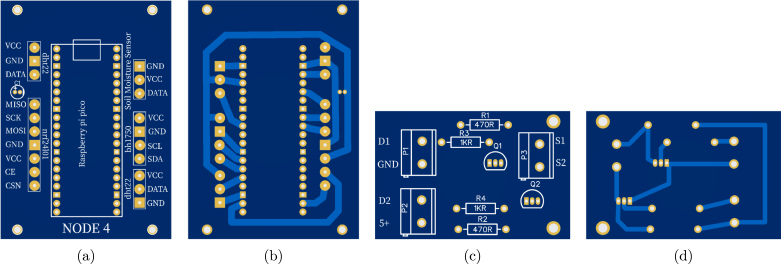
PCB Layout examples: (a) Node 4 silkscreen/component view, (b) Node 4 copper layer (bottom), (c) Logic Level Shifter silkscreen/component view, (d) Logic Level Shifter copper layer (bottom).

The system utilizes modular single-sided PCBs, designed to be manufacturable using standard low-cost techniques such as toner transfer chemical etching. Fig. 8 illustrates the layout: Node 4 acts as a representative example of the sensor nodes, featuring dedicated zones for the MCU and sensor inputs, while the Level Shifter board demonstrates the isolation required for actuator control.

For full reproducibility, the complete list of manufacturing files (traces and silkscreen) and the step-by-step toner transfer etching procedure are provided in Appendix G.1.

### 5.6 Hardware assembly

This section details the assembly of **Node 4** as a representative example. Comprehensive step-by-step soldering instructions, wire lengths, pinout charts, and assembly details for the remaining nodes (Nodes 0-3) are provided in the supplementary material (**Appendix G.2**).

#### 5.6.1 Sensor node assembly (Representative node 4)

**Fig. 9:**
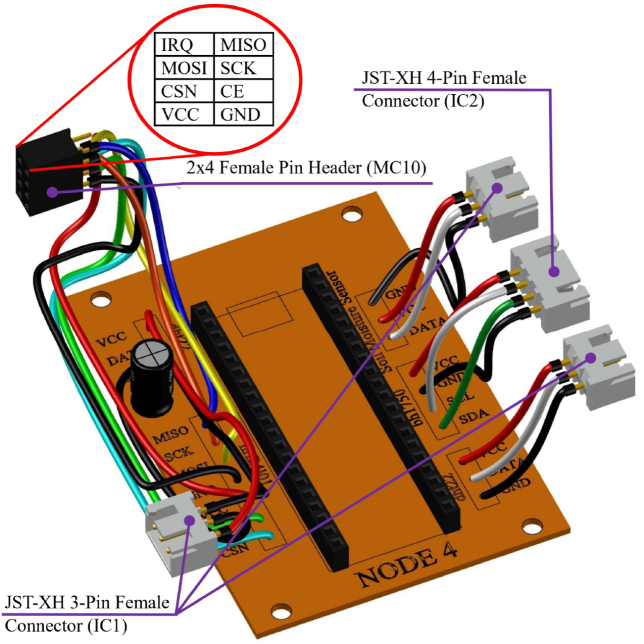
Assembled PCB for Node 4. Note the placement of the MCU headers and JST connectors for peripheral stability.

The board is populated with 1 × 20 female headers (MC01) to house the Raspberry Pi Pico (RP02), allowing for modular replacement. Peripheral connections for sensors and power are established using JST-XH connectors (IC01, IC02). The nRF24L01 radio module and external sensors utilize flexible wiring to facilitate optimal antenna positioning within the enclosure, as illustrated in Fig. 9.

#### 5.6.2 Logic inverter, actuation control, and LoRa expansion

**Fig. 10:**
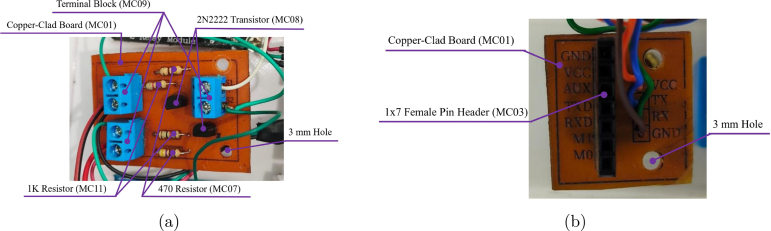
Auxiliary control boards: (a) 3.3V–5V Logic Inverter/Level Shifter, (b) LoRa expansion board.

**Logic Inverter and Level Shifter Design:** To address the voltage incompatibility between the 3.3 V logic of the Raspberry Pi Pico and the 5 V relay modules, a dedicated PCB was designed (Fig. 10(a)). It converts 3.3 V GPIO signals to 5 V compatible levels using NPN transistors (2N2222, MC08). The relay modules used (AC04, AC05) are active-low (trigger on 0 V); the transistor circuit inverts the active-high output of the Pico. It protects the microcontroller from inductive transients generated by actuators (fans, solenoid valve).

**Design Calculations:** See (**Appendix A**) for transistor biasing equations.

Additionally, the Node 0 is expanded with a custom LoRa interface board utilizing a 1 × 7 header layout (see Fig. 10(b)) to accommodate the long-range transceiver, ensuring dedicated power lines and SPI bus integrity.

### 5.7 Enclosure integration and power subsystem

**Fig. 11:**
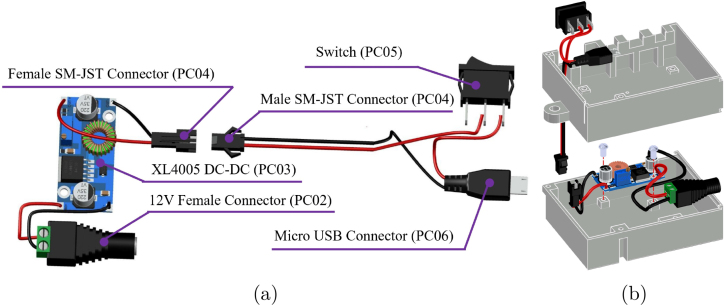
Node Power Supply System: (a) DC–DC conversion and distribution components, (b) Physical placement within the enclosure.

A unified power supply subsystem (see Fig. 11) was implemented across all nodes, based on a high-efficiency step-down converter (XL4005, PC03) that converts the external 12 V input to a stable 5 V bus for the microcontroller. Input power is delivered via a standard DC jack (PC02) and controlled by a rocker switch (PC05). While the core topology is identical, Node 2 includes a parallel 12 V rail to power the industrial pH sensor, and Node 1 adapts the wiring for 5 V sensor requirements. Detailed wiring diagrams for these variations are provided in (**Appendix D**).

The PCBs are mounted onto the enclosure base using M3 hexagonal spacers (GM02) to ensure electrical isolation and mechanical rigidity (see installation details in **Appendix G.3**). Once the mechanical structure is secured, the communication modules (nRF24L01, WC02) and the Raspberry Pi Pico are inserted into their headers.

**Fig. 12:**
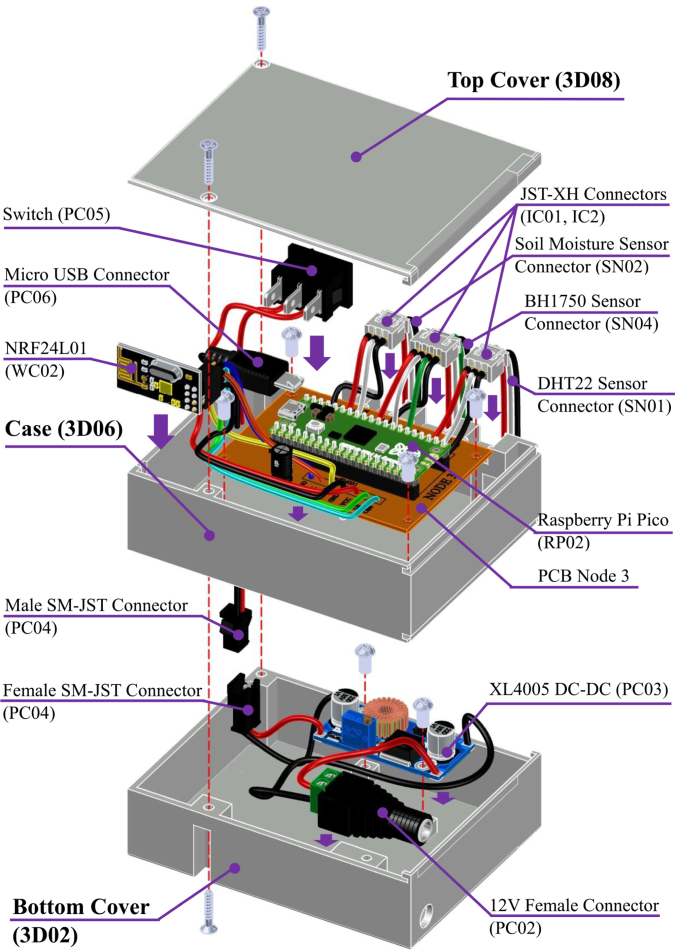
Internal view of the fully assembled Node 3, showing the integration of the power subsystem, PCB, and communication modules.

**Final Integration:** Fig. 12 illustrates the fully assembled internal view of a standard node. For the Gateway (Node 0), the assembly additionally includes the LoRa transceiver wiring and the RJ-45 interface (IC03) for RS-485 connectivity, routed through the designated chassis ports.

Critical verification steps for voltage regulation (step-down converter) and sensor addressing are listed in **Appendix G.9**.

#### 5.7.1 Actuation control unit integration

The actuation subsystem is consolidated within a dedicated enclosure to isolate high-current switching components from the sensitive sensor nodes. This unit houses the Logic Level Shifter PCB alongside the relay modules (AC04, AC05), which are responsible for driving the solenoid valve (AC02) and ventilation fans (AC03).

**Fig. 13:**
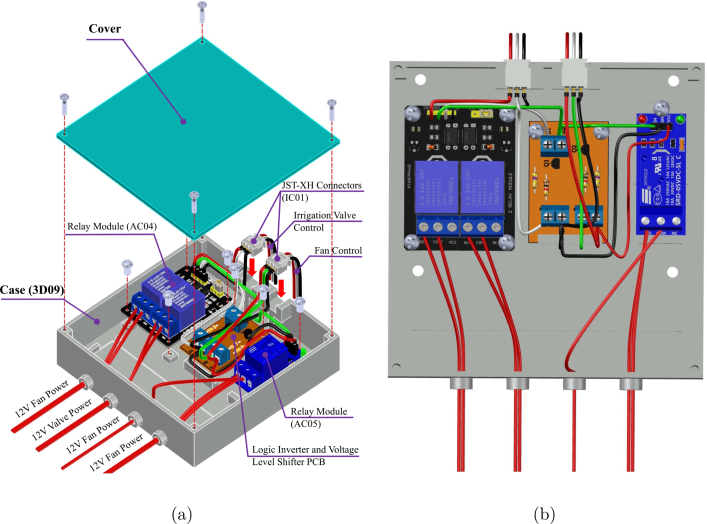
Actuator Control Enclosure integration: (a) Internal layout of relays and logic board, (b) Sealed unit with acrylic cover.

As shown in Fig. 13, the internal arrangement prioritizes cable management and thermal dissipation. The assembly is sealed with a transparent acrylic cover (GM06), allowing for visual inspection of the relay status LEDs during operation while maintaining environmental protection against dust and moisture.

### 5.8 Main base to gateway interface

**Fig. 14:**
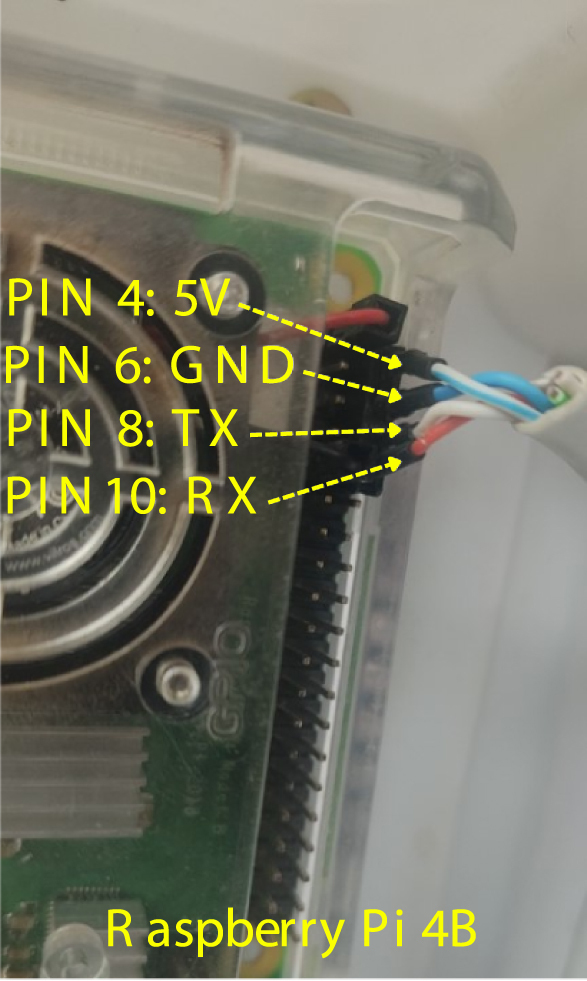
Pinout diagram for the Raspberry Pi 4B interface connecting to the Gateway Node via UTP.

Reliable full-duplex communication between the Main Base (Raspberry Pi 4B) and the Gateway (Node 0) is established via a hardwired UART link utilizing standard UTP cabling. This interface was designed to carry both power (5 V bus) and data signals (TX/RX) over a single robust connection, minimizing signal degradation in the field. The physical layer adheres to a custom pinout mapping based on the T-568B connector standard (Fig. 14), ensuring that the Raspberry Pi 4B (RP01) GPIOs correctly interface with the microcontroller logic of the Gateway. For detailed mounting instructions refer to **Appendix G.5**.

#### 5.8.1 External lora module enclosure

**Fig. 15:**
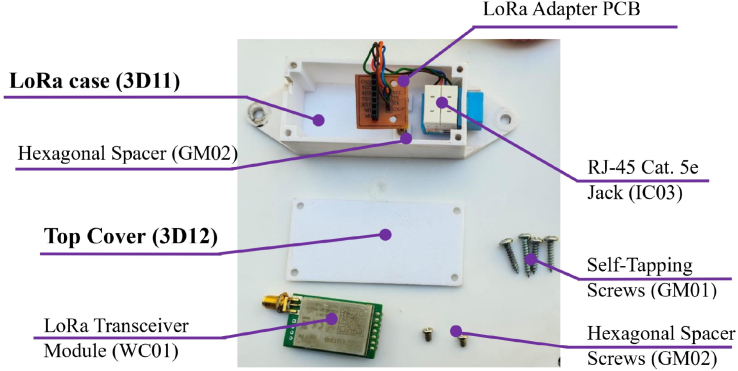
Components for cover case LoRa.

To maximize RF range and ensure line-of-sight connectivity, the LoRa transceivers are housed in dedicated external enclosures (Fig. 15), separate from the main processing units. This modular design isolates the RF components from the digital noise of the Raspberry Pi and allows for high-elevation mounting.

The enclosure integrates a custom adapter PCB secured via hexagonal spacers to ensure mechanical stability. Connectivity is established through an RJ-45 interface, which carries power and SPI data from the main node. The assembly is designed to be weather-resistant, with the LoRa module positioned near the top cover to facilitate the connection to the external antenna.

For details of the antenna connector (915 MHz) and the coaxial cable assembly, please refer to (**Appendix G.5.1**).

#### 5.8.2 Sensor integration and environmental hardening

Given the high humidity conditions inside the greenhouse and the exposure to soil electrolytes, standard commercial sensors required specific waterproofing and mechanical reinforcement modifications to prevent corrosion and data drift. For a detailed illustrated guide on sensor waterproofing refer to (**Appendix G.6**) and for actuators and auxiliary sensors (**Appendix G.7**)

### 5.9 Final system integration and field deployment

The final deployment configuration establishes the physical interfaces between the processing units, communication modules, and external sensors.

**Fig. 16:**
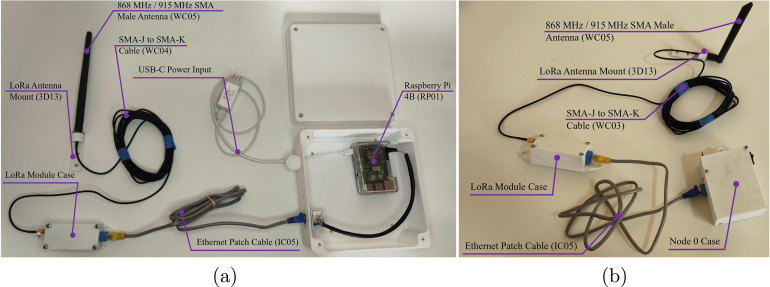
Final integration of the central communication infrastructure. (a) Main Base Assembly, (b) Node 0 Integration.

The Main Base connects the LoRa transceiver to the Raspberry Pi 4B via the shielded UTP interface described previously, secured within a junction box (GM07) for dust protection (see Fig. 16(a)). Similarly, Node 0 is powered via a 12 V source and bridged to its external RF unit via the RJ-45 link, as shown in Fig. 16(b).

**Fig. 17:**
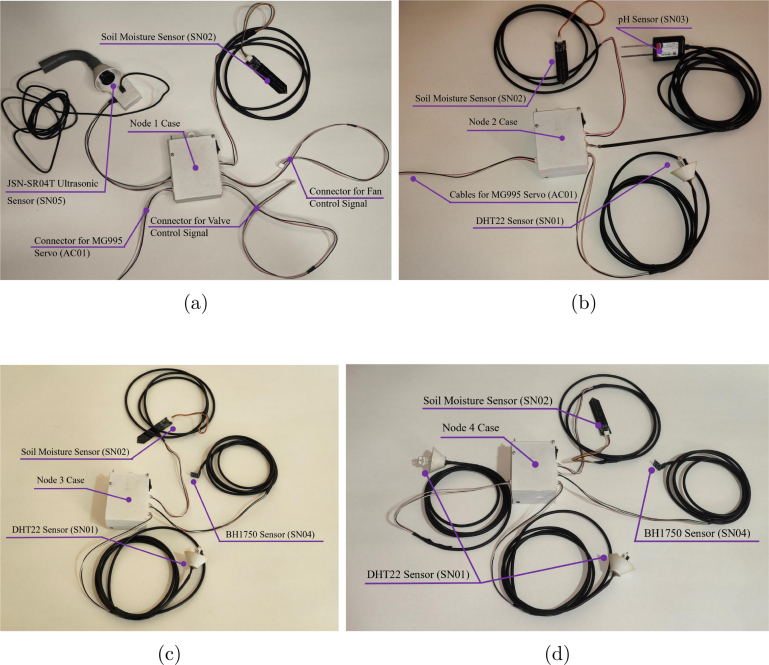
Sensor connectivity and final configuration per node: (a) Node 1, (b) Node 2, (c) Node 3, (d) Node 4.

The distributed nodes (1-4) utilize external waterproof connectors to interface with the environmental sensors (temperature, humidity, soil moisture, pH, light, and distance probes). This modular design allows for rapid sensor replacement in the field without compromising the internal electronics enclosure. The specific sensor load out for each node is detailed in Fig. 17.

### 5.10 Software architecture and deployment

This section details the logical implementation of the distributed sensor network. The complete source code, developed in MicroPython for the edge nodes and Python 3 for the Main Base, is available in the OSF repository ( https://doi.org/10.17605/OSF.IO/APHCV).

The deployment strategy relies on the scripts listed in Table 1. It is critical to note that the Raspberry Pi Pico (RP02) bootloader automatically executes the file named main.py upon power-up (5 V). Consequently, the node-specific scripts must be renamed to main.py during the flashing process. The modules nrf24l01reg.py (PY07) and dht.py (PY08) function as essential driver libraries and must reside in the root directory of the microcontroller.

**Fig. 18:**
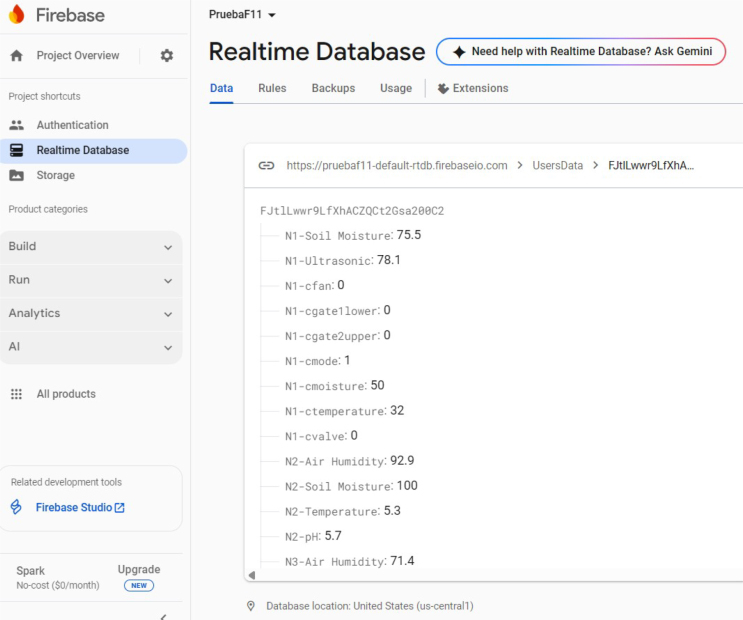
Configuration interface for the Firebase Realtime Database project.

**Fig. 19:**
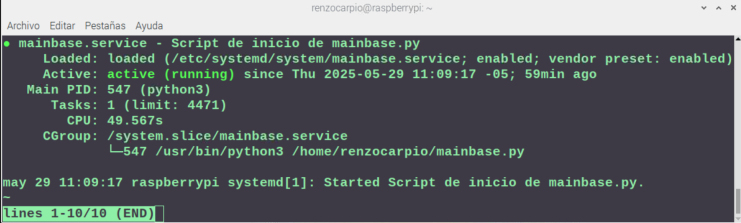
System resilience verification: main_base.py running as a background service on the Raspberry Pi 4B.

The system configuration is divided into two logical layers:

• **Main Base:** The logic for the Raspberry Pi 4B (RP01) is encapsulated in the main_base.py script (PY01). Network stability is enforced by configuring a static IP address.
  Data synchronization is managed via the Google Firebase Realtime Database. The cloud integration requires the initialization of a project console as depicted in Fig. 18, ensuring that the database credentials match the parameters defined in the source code.
  To guarantee system resilience particularly crucial in rural areas with unstable power grids the control script is configured to execute as a persistent background service (daemon). This operational mode, illustrated in Fig. 19, ensures automatic recovery and re-initialization of the data acquisition loop approximately 10 s after a system reboot or software exception.
• **Node:** Each distributed node requires a specific firmware configuration based on its hardware role. The complete file mapping table, detailing the specific main.py script and driver libraries required for each node ID, is provided in **Appendix G.8** in Table 7

### 5.11 Software implementation details

This section presents the specific algorithms developed to execute the logic described in the architecture (Section 5.1). The following snippets illustrate the MicroPython implementation of the serialization protocol and the cloud synchronization routine.

#### 5.11.1 Data serialization implementation

To strictly adhere to the 32-byte payload limit of the nRF24L01 described in Section 5.1, the nodes employ a positional string formatting method. Code Listing 1 demonstrates how Node 2 packs four sensor variables into a fixed-length string, eliminating the overhead of JSON parsing.

**Figure:**
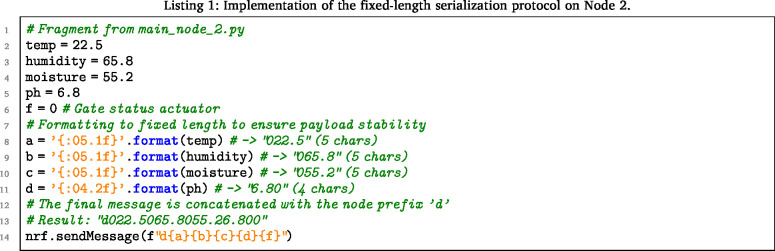

#### 5.11.2 Cloud synchronization and persistence logic

The Main Base ensures data integrity through the dual-write strategy. Code Listing 2 shows the retrieval of the control vector (cs), while Listing 3 illustrates the simultaneous logging to the local SD card and the Firebase cloud.

**Figure:**
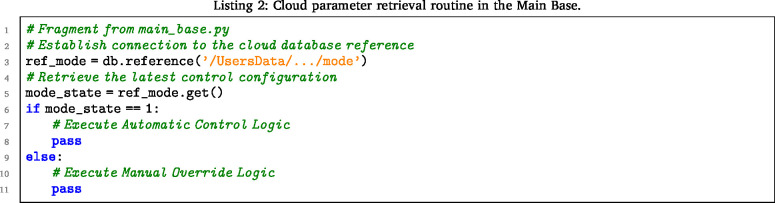

Once the sensor data is collected and processed, the system ensures data persistence through a dual-write strategy: data is uploaded to the cloud for visualization and simultaneously appended to a local log file.

**Figure:**
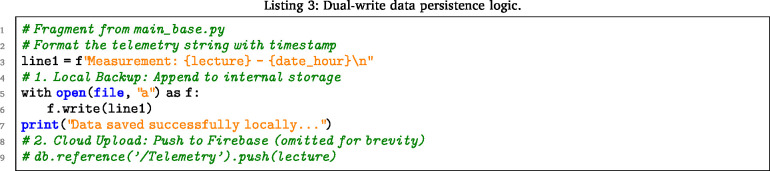

#### 5.11.3 Control signal (CS) structure

**Fig. 20:**
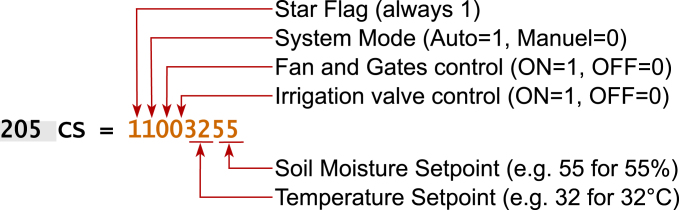
Structure of the Control Signal (CS) frame used to synchronize the Main Base with the distributed nodes.

As defined in the control loop, the Control Signal (CS) compresses the actuator states into a single integer. Fig. 20 details the bitwise mapping used by the nodes to decode this vector.

#### 5.11.4 nRF24L01 configuration and collision avoidance

The nRF24L01 transceivers are configured with the following parameters to ensure reliable communication within the greenhouse (see Table 3):

**Interference Mitigation:** Channel 60 (2460,MHz) was selected to avoid congestion from Wi-Fi channels 1 and 6 (centered at 2412 and 2437,MHz). Although this frequency falls within the bandwidth of Wi-Fi channel 11, a spectral scan of the greenhouse deployment site confirmed that this specific band was clear of active traffic, allowing for robust link quality.

**Table 3:** nRF24L01 configuration parameters.

Parameter	Value
RF channel	60 (2460 MHz)
Data rate	1 Mbps
Payload size	32 bytes (fixed)
CRC	16-bit enabled
Auto-ACK	Enabled
Auto-retransmit count	15 attempts
Auto-retransmit delay	1500μs
Network address	0 × 756E736100 (“unsa”)

## 6 Operation instructions

The PyLoGreen system is designed for autonomous “plug-and-play” operation. This section distinguishes between the daily startup routine and the sporadic maintenance tasks required for system longevity.

### 6.1 Standard startup sequence

For daily operation, no code execution is required. The system follows this automatic sequence upon connecting the 12 V DC power supply:

1. **Power Stabilization:** Voltage regulators stabilize the 5 V bus; sensor nodes boot immediately.
2. **Gateway Synchronization:** Node 0 enters a standby state to prevent data loss, waiting for the handshake signal.
3. **Mesh Link Establishment:** The Main Base (Raspberry Pi 4B) boots, launches the background service, and transmits the control frame. Node 0 detects this signal and enables the wireless network.
4. **Continuous Acquisition:** The system enters the infinite polling loop.

### 6.2 Monitoring interfaces

System status can be verified through two channels:

• **Online:** Real-time visualization via the Firebase Console.
• **Offline:** Inspection of the local telemetry log stored on the Main Base SD card (accessible via SSH).

#### 6.2.1 LoRa frequency configuration (one-time event)

The LoRa modules ship with a default 868 MHz configuration. Before assembly, they must be permanently reconfigured to 915 MHz to match the antenna hardware.

• Set pins M0 and M1 to HIGH. The configuration command sent via UART is 0xC0 0x00 0x00 0x1A 0x35 0x44.
• Then follow and execute the configuration script provided in PY09 once. No further software intervention is required during normal operation.

#### 6.2.2 Sensor calibration

To ensure accurate Relative Moisture Index readings, calibrate the capacitive sensor against the specific soil type:

1. **Dry Reference (0%):** Insert the sensor into oven-dried soil to measure the baseline dry ADC value ($ADC_{dry}$). Update the max_moisture constant in the firmware.
2. **Saturation Reference (100%):** Insert the sensor into fully saturated soil (mud state) to measure the wet ADC value ($ADC_{wet}$). Update the min_moisture constant.

The firmware maps the operational readings ($ADC_{read}$) using the linear inversion formula shown in Eq. (1), which accounts for the voltage drop associated with increased dielectric permittivity:

(1) $$\text{\%}H=\left(\frac{ADC_{dry}-ADC_{read}}{ADC_{dry}-ADC_{wet}}\right)\times 100$$

**pH Sensor Verification:** Unlike generic probes, the industrial RS-PH-*-TR-1 transmitter is factory-calibrated. Verification against the expected local soil pH range (6.5–7.5) is sufficient to confirm operational integrity; re-calibration is only necessary if significant drift is observed.

### 6.3 Operational safety precautions

• **Power Limits:** Ensure input voltage remains strictly within 11.5–14.0 V DC to prevent thermal shutdown of the regulators.
• **Moisture Integrity:** While sensors are waterproof, the main processing enclosures are splash-resistant (IP65). Do not submerge the central nodes.
• **Hot-Swapping:** Always disconnect main power before opening enclosures or swapping sensors to prevent GPIO short circuits.

### 6.4 Software requirements

The following software is required:

• The required operating system is Raspberry OS 64-bit.
• Thonny 4.1.7
• Visual Studio Code 1.101.2
• Python 3.9
• EasyEDA 2.2.39.2
• AutoCAD 2024 (Blender 4.0 can also be used)
• UltiMaker Cura V5.7.0
• RealVNC Viewer 7.12.1 (optional for viewing the Raspberry OS interface on Windows)
• Google Earth Pro V7.3.6.9796

## 7 Validation and characterization

The validation of the system focused on demonstrating operational robustness and the reliability of the data acquisition pipeline in a challenging, real-world environment. Field tests were conducted in a greenhouse in Juliaca, Peru, ensuring the design was validated under the high-Andean tundra conditions for which it was developed.

It is important to note that while the system was first commissioned in mid-2024, the primary dataset presented herein corresponds to a continuous uninterrupted period in August 2025. Furthermore, specific stress validations, such as the maximum range test, were conducted five months post-acquisition, ensuring that the results reflect the performance of hardware that has endured over a year of cumulative exposure to the high-Andean tundra conditions

### 7.1 LoRa communication range and reliability

**Fig. 21:**
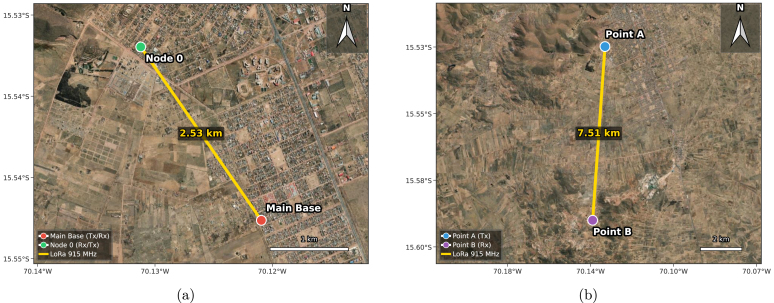
Geographic location of the LoRa links in Juliaca, Peru. (a) Operational link between the Main Base and Node 0 with partial Line-of-Sight at 2.53 km. (b) Maximum range validation test (7.51 km) between Point A (Tx) and Point B (Rx).

A key objective was to confirm that the hardware, including the 3D-printed PETG enclosures and external antennas, could maintain a stable long-range LoRa link under adverse environmental conditions (low temperatures, high UV). The geographic arrangement of the test scenarios is illustrated in Fig. 21.

#### 7.1.1 Operational link analysis (2.53 km)

**Fig. 22:**
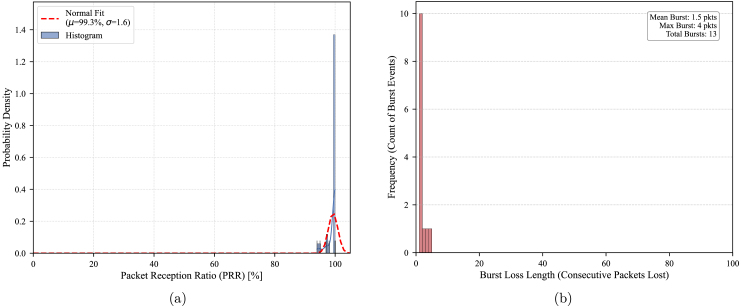
Link validation at 2.53 km (Urban/Rural Interface). (a) PRR Distribution showing a stable 99.3% average, (b) Burst Loss Analysis where minimal consecutive packet losses indicate a robust link.

The primary link (Main Base to Node 0) operated with partial line-of-sight. As shown in Fig. 22, the connection was highly stable, achieving a mean Packet Reception Ratio (PRR) of **99.3%** with a low variance of 1.6%. The burst loss analysis confirms channel coherence; consecutive packet losses were rare (maximum of 4 packets), ensuring continuous data availability for agricultural monitoring.

#### 7.1.2 Maximum range stress test (7.51 km)

**Fig. 23:**
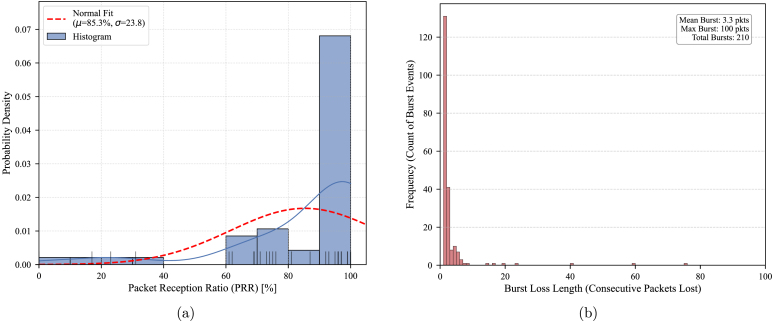
Link validation at 7.51 km (Deep Rural/NLOS). (a) PRR Distribution where average PRR drops to 85.3% near sensitivity limits, (b) Burst Loss Analysis where longer consecutive losses indicate multipath fading.

To determine the hardware limits, a long-range test was conducted (Fig. 23). While an effective average PRR of **85.3%** was maintained, the variance increased significantly to 23.8%. The burst analysis revealed instances of 100% packet loss, indicating deep fading effects and temporary obstructions typical of Non-Line-of-Sight (NLOS) conditions at this distance. The firmware implementations for the transmitter and receiver are available in PY10 and PY11.

### 7.2 Physical deployment

**Fig. 24:**
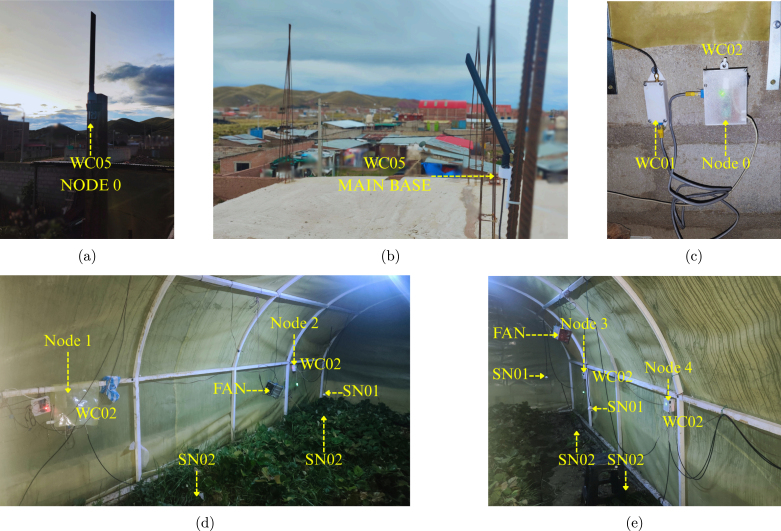
On-site system deployment in Juliaca. (a) High-gain antenna installation for Node 0. (b) Base station antenna mast. (c) External view of the Node 0 enclosure and weatherproofing connections. (d-e) Sensor nodes installed in the greenhouse soil for real-time monitoring of crop conditions.

The theoretical network topology detailed in the deployment diagram (Fig. 4) is validated by the actual on-site installation presented in Fig. 24. These images confirm the mechanical integration of the sensor nodes within the active crop canopy and the structural stability of the elevated antenna mounts required for the LoRa link.

### 7.3 Power consumption analysis

The power consumption of the PyLoGreen system was evaluated at each node to ensure energy efficiency during long-term agricultural monitoring. Measurements were performed using an FNIRSI 1014D digital oscilloscope across a 1Ω shunt resistor on the 12 V supply rail.

Total power is calculated as the product of the bus voltage (12 V) and the measured average current ($I_{avg}$), which inherently accounts for the switching efficiency of the DC–DC step-down converters.

**Fig. 25:**
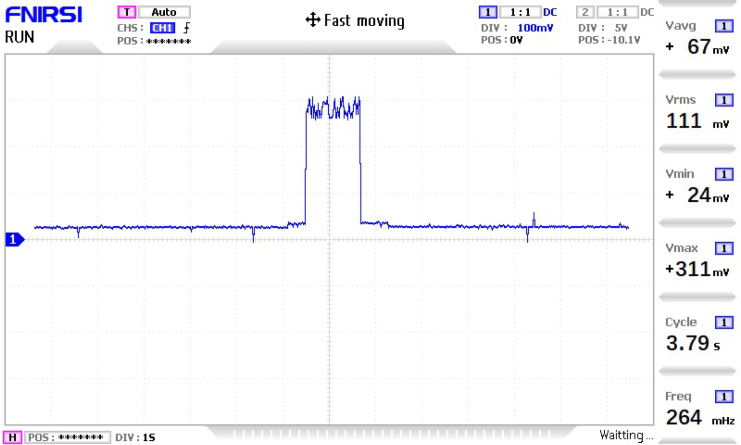
Current consumption profile for Node 0 (Gateway). The distinct peaks correspond to the simultaneous TX operations of the LoRa and nRF24L01 modules.

As shown in Fig. 25, Node 0 exhibits the highest peak current (311 mA) due to RF transmission bursts. In contrast, the sensor nodes (1-4) exhibit significantly lower average consumption. Detailed oscillograms characterizing the switching noise and load profiles for these nodes are provided in **Appendix H**.

Table 4 summarizes the experimental results.

**Table 4:** Theoretical and measured current consumption at 12 V per node.

Node ID	Key components	Theoretical (mA)		Measured (mA)		
		Min	Max	Min	Max	Avg.
Node 0	nRF24L01,	25	331	24	311	67
	LoRa E32					

Node 1	nRF24L01, JSN-SR04T,	32	550	36	367	161
	Soil Sensor, Relays					

Node 2	nRF24L01, DHT22,	48	300	–1	251	49
	Soil Sensor, pH Probe					

Node 3	nRF24L01, DHT22,	28	45	–1	57	23^2^
	BH1750, Soil Sensor					

Node 4	nRF24L01, 2 × DHT22,	29	46	–1	113	29^2^
	BH1750, Soil Sensor					

1 Instantaneous minimums obscured by DC-DC switching noise.

2 Value reported as RMS for stability.

### 7.4 System dynamics and functional validation

To address the need for detailed functional validation and to clarify the specific interactions between control variables, we analyzed a high-activity 3-day window (August 13–15, 2025). Unlike the aggregated density maps presented in the subsequent section, which demonstrate long-term stability, these time-series plots validate the immediate responsiveness of the control loops and the physical coherence of the multi-sensor data.

#### 7.4.1 Actuator response and latency (Node 1)

**Fig. 26:**
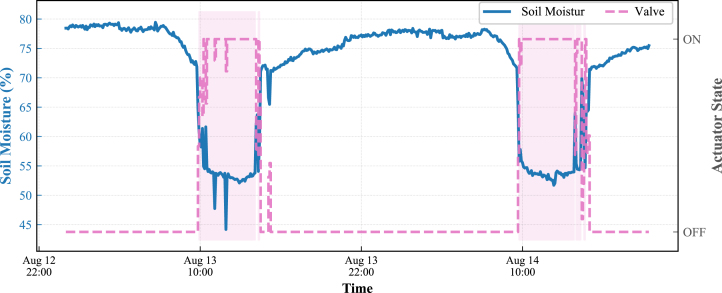
Functional validation of Node 1 irrigation logic. The time series confirms the correct execution of the hysteresis cycle, showing the correlation between valve activation durations and soil moisture recovery.

Fig. 26 illustrates the precise response of the irrigation control loop at Node 1. The time series demonstrates a direct correlation between actuator activity (Fan and Valve states, shown in the bottom panel) and the physical response in soil moisture levels (top panel).

It is observed that the valve activates precisely when soil moisture drops below the lower threshold. Due to the slow dynamics of the drip irrigation system, the valve remains in the active state for a sustained period until the moisture level gradually recovers to the upper setpoint. This behavior confirms the correct execution of the autonomous hysteresis control logic, ensuring adequate water absorption without overshoot.

#### 7.4.2 Thermodynamic consistency and substrate stability (Node 2)

**Fig. 27:**
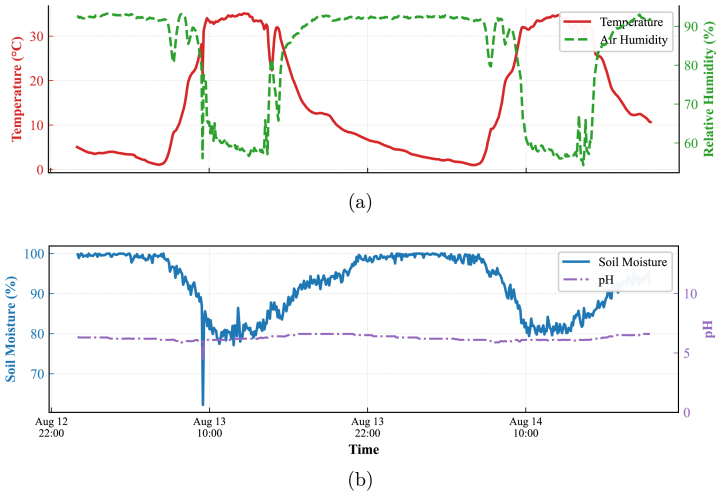
Signal integrity verification at Node 2. (a) Atmospheric Dynamics (Temp vs Humidity) showing that digital sensors correctly capture the inverse correlation, (b) Substrate Dynamics (Soil Moisture vs pH) where the analog pH interface maintains signal stability despite soil moisture swings, verifying channel isolation.

The environmental monitoring performance of Node 2 is detailed in Fig. 27. Fig. 27(a) demonstrates the ability of the system to accurately track the physical inverse relationship between air temperature and relative humidity, confirming the responsiveness of the digital acquisition bus. Crucially, Fig. 27(b) validates the electrical stability of the analog front-end. Despite significant variations in soil moisture (which alters the electrical conductivity of the medium), the pH sensor output remains stable with no induced noise. This confirms that the hardware design effectively isolates the high-impedance pH probe from cross-talk interference, ensuring reliable data acquisition independent of soil water content.

**Fig. 28:**
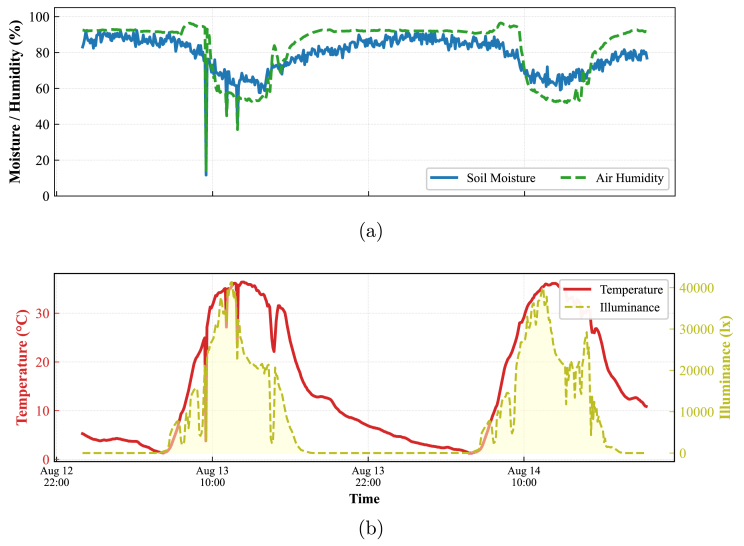
Multi-protocol validation at Node 3. (a) Moisture Comparison showing the correlation between analog and digital moisture readings, (b) Energy Dynamics where the temporal lock between Solar Radiation ($I^{2}$C) and Temperature (1-Wire) proves effective firmware task scheduling and protocol coexistence.

**Fig. 29:**
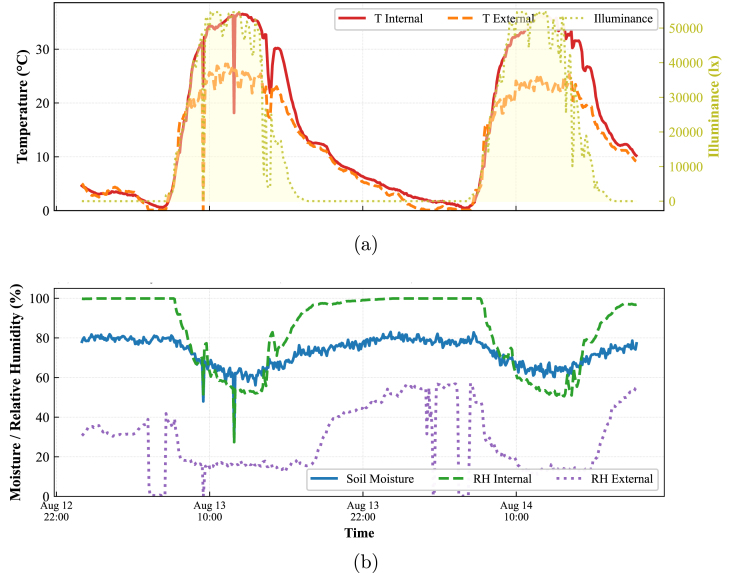
Simultaneous environmental monitoring at Node 4. (a) Superposition of Internal vs. External temperature profiles correlated with solar radiation. (b) Comparison of humidity levels, verifying the capability of the system to log differential data streams.

#### 7.4.3 Multi-protocol acquisition and bus arbitration (Node 3)

Fig. 28 validates the robustness of Node 3 in managing concurrent data streams from heterogeneous communication interfaces ($I^{2}$C, 1-Wire, and Analog).

The critical validation lies in the temporal alignment shown in Fig. 28(b). The plot demonstrates a precise synchronization between the Illuminance peaks ($I^{2}$C bus) and the subsequent rise in Air Temperature (1-Wire bus). Since the 1-Wire protocol requires strict timing slots compared to the faster $I^{2}$C interface, this perfect correlation confirms that the firmware successfully performs bus arbitration without blocking delays or data loss, maintaining signal coherence across physically different sensors.

#### 7.4.4 Comparative microclimate analysis (Node 4)

Fig. 29(a) validates the ability of the system to monitor internal and external environments simultaneously. Fig. 29(b) presents the superposition of the internal (red) and external (blue) temperature profiles, alongside solar radiation (yellow).

Rather than plotting a calculated differential curve, the direct visual comparison of the two temperature profiles reveals the thermal dynamics of the greenhouse. The system successfully captures how the internal temperature tracks the external ambient conditions with high fidelity. The correlation between the illuminance peaks and the thermal response of both sensors confirms that the distributed network is temporally synchronized and capable of resolving the specific microclimatic conditions inside the structure versus the open field.

### 7.5 Long-term system stability and reliability

Complementing the functional validation, operational data collected continuously from July 28 to August 26, 2025, was analyzed to verify the long-term robustness of the hardware. To address the visual complexity of plotting 30 days of raw time-series data, this analysis utilizes two-dimensional density maps. In these visualizations, high-density regions (hotspots) represent recurrent system states, allowing for the verification of sensor consistency and timing precision without the clutter of transient noise.

#### 7.5.1 Actuation reliability and RTC precision (Node 1)

**Fig. 30:**
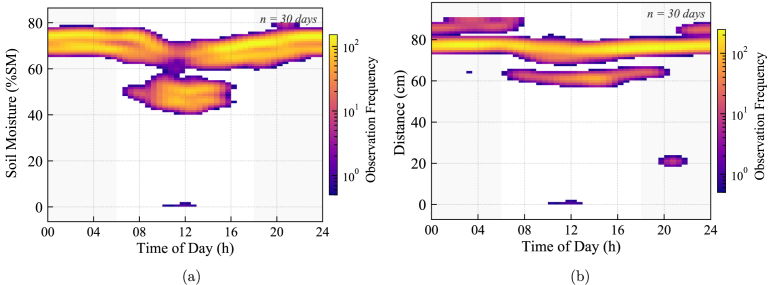
Long-term stability analysis of Node 1 (30 days). (a) Soil moisture remains bounded within the control setpoints. (b) Water level data shows repetitive depletion patterns (vertical clustering), confirming the precision of the RTC-based scheduling.

The 30-day density analysis for Node 1 (Fig. 30) validates the stability of the Real-Time Clock (RTC) and the hysteresis control loop.

In Fig. 30(b), the ultrasonic water level data does not appear as random noise but forms distinct vertical clusters. These high-density bands correspond to the repetitive tank depletion events occurring at strict time intervals (08:00, 12:00, 16:00) every day. The sharpness of these temporal clusters confirms that the microcontroller executed over 90 autonomous irrigation cycles with negligible timing drift over the month. Similarly, Fig. 30(a) shows that soil moisture readings are strictly confined within the programmed hysteresis limits, proving that the control loop effectively prevents both waterlogging and substrate desiccation.

**Fig. 31:**
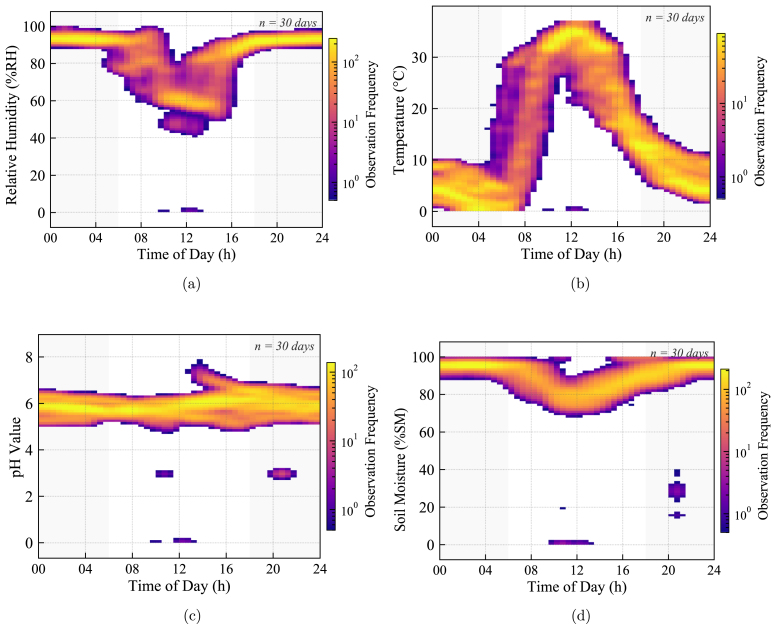
Analog signal integrity at Node 2 (30 days): (a) Internal Relative Humidity, (b) Internal Air Temperature, (c) Soil pH Stability, and (d) Soil Moisture Content. The sharply defined density regions confirm consistent ADC performance and high signal-to-noise ratio; substrate readings remain clustered, validating the stability of the analog front-end.

**Fig. 32:**
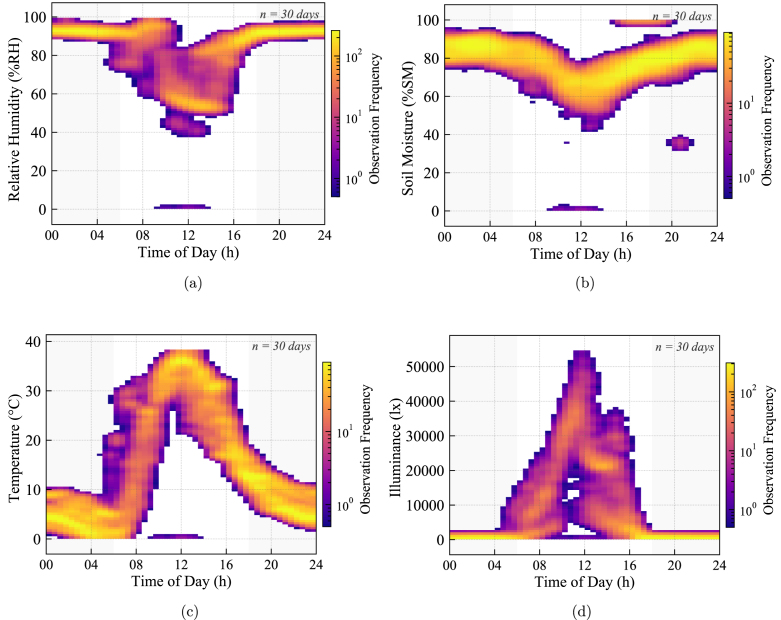
Protocol stability at Node 3 (30 days): (a) Relative Humidity (1-Wire), (b) Soil Moisture (Analog), (c) Air Temperature (1-Wire), and (d) Illuminance (I${}^{2}$C). The continuous density patterns verify reliable data acquisition across heterogeneous interfaces (1-Wire, Analog) without timing conflicts. The smooth density gradients in light and temperature data confirm correct I${}^{2}$C/1-Wire bus arbitration and long-term firmware stability.

**Fig. 33:**
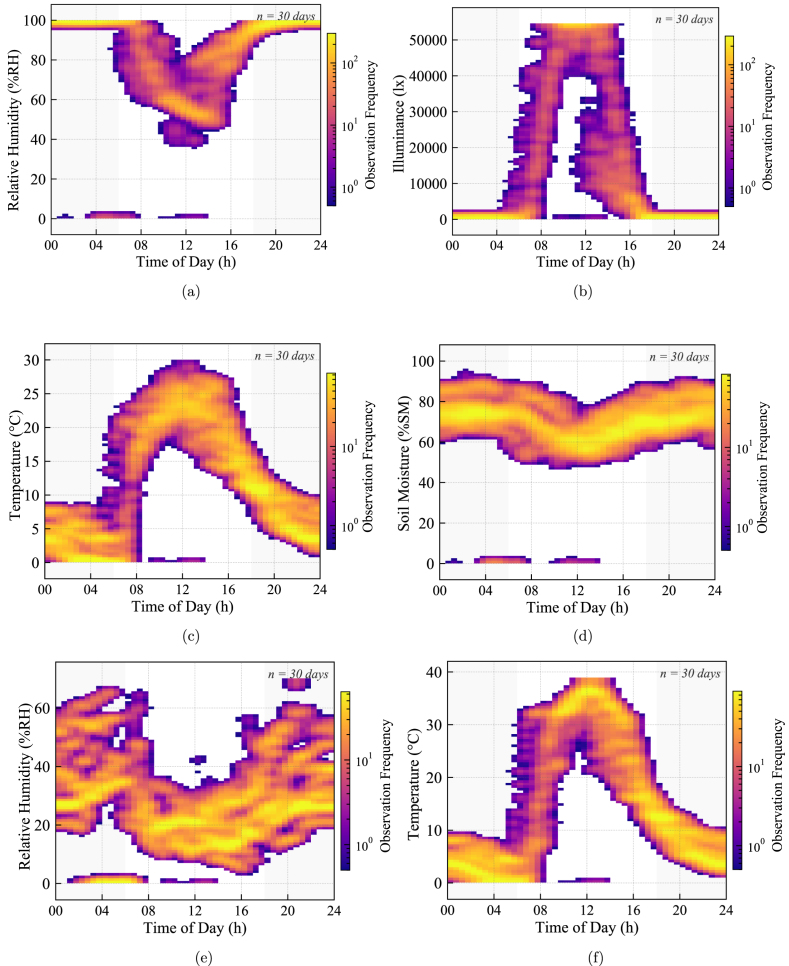
Differential profiling at Node 4 (30 days): (a) Internal Relative Humidity, (b) External Illuminance, (c) External Ambient Temperature, (d) Internal Soil Moisture, (e) External Relative Humidity, and (f) Internal Air Temperature. The contrast between dispersed or chaotic external signals (c, e) and tightly clustered internal data (a, d, f) supports the ability of the system to capture the buffering effect of the greenhouse structure while maintaining signal integrity across contrasting environments.

Regarding the substrate monitoring, Fig. 30(a) validates the Relative Moisture Index (RMI) calibration. The high-density hotspots are strictly confined between the programmed hysteresis limits, proving that the empirically determined calibration constants ($ADC_{dry}=19000$ and $ADC_{wet}=9500$) correctly captured the dynamic range of the substrate. The absence of vertical data dispersion outside these bounds over the 30-day period confirms that the sensor drift was minimal (under 3%) and that the two-point linear calibration remains stable for long-term greenhouse automation without requiring manual offsets.

#### 7.5.2 Analog signal integrity and digital bus stability (Nodes 2 & 3)

The aggregated density analysis for Node 2 (Fig. 31) and Node 3 (Fig. 32) provides conclusive evidence regarding the electrical stability of the sensor interfaces over long-term operation.

For Node 2, the substrate sensors (pH and Moisture) exhibit highly localized density clusters rather than dispersed noise clouds. This sharp distribution confirms the precision of the Analog-to-Digital Converter (ADC) and the effectiveness of the hardware filtering design; there is no evidence of power supply ripple or ground loop interference affecting the low-voltage analog signals.

Similarly, Node 3 validates the robustness of the digital communication stacks. The smooth, continuous gradients observed in the $I^{2}$C illuminance data (Fig. 32(d)) and the 1-Wire temperature readings (Fig. 32(c)) confirm that the firmware correctly manages bus arbitration over millions of read cycles. The absence of scattered outliers or flat-line artifacts indicates zero packet loss and verifies that the system operates without memory leaks or bus lock-ups.

The density analysis of Fig. 31(c) confirms that the sensor output remained stable and clustered within this valid agronomic range during the 30-day deployment, showing no signs of the signal drift typically associated with uncalibrated sensors.

#### 7.5.3 Differential environmental profiling and network coherence (Node 4)

Finally, Node 4 serves as a benchmark for differential environmental analysis, measuring both internal (controlled) and external (ambient) conditions simultaneously. Fig. 33(a) reveals a distinct contrast in statistical behavior that validates the dynamic range of the system.

The internal metrics, such as Soil Moisture (Fig. 33(d)) and Internal Temperature (Fig. 33(f)), exhibit highly concentrated density hotspots, identical in pattern to Nodes 1, 2, and 3. This consistency across distributed nodes confirms that the wireless network maintains data integrity without packet corruption.

Conversely, the external metrics-Illuminance (Fig. 33(b)) and Ambient Temperature (Fig. 33(c)) display wide, diffuse density gradients. This visual dispersion accurately represents the high variance of the High-Andean weather. The fact that the microcontroller simultaneously captures the stability of the internal microclimate and the volatility of the external environment on the same timeline confirms the capability of the hardware to perform reliable differential profiling.

### 7.6 Capabilities, limitations, and future considerations

The PyLoGreen system was designed and validated to balance performance, cost, and replicability. The following points characterize its operational capabilities, acknowledge current constraints, and outline the roadmap for scalability.

#### 7.6.1 Capabilities and design rationales

• **Hybrid Communication Architecture:** The system effectively combines LoRa technology for long-range links (validated at 2.53 km in field tests) with an nRF24L01-based local sensor network, creating a cost-effective and functionally optimized communication pipeline.
• **Strategic Protocol Selection (P2P LoRa):** The decision to implement Point-to-Point (P2P) LoRa instead of LoRaWAN was driven by the specific constraints of the high-Andean deployment. Unlike LoRaWAN, which requires expensive gateway infrastructure and cloud dependency, P2P offers determinism and offline autonomy. This allows for predictable packet timing critical for actuator control and ensures continuous operation during internet blackouts common in rural Peru.
• **Low-Cost and Open-Source Design:** With a total hardware cost of approximately $367, the project is highly accessible. Its implementation using open-source hardware (Raspberry Pi) and software (MicroPython) ensures easy replication and community-driven adaptation.
• **Resilience and Autonomous Operation:** The system is engineered for unattended deployment, incorporating software watchdog mechanisms in the sensor nodes for automatic recovery from errors and the capability for the Main Base to operate offline, ensuring continuous data logging even without an internet connection.
• **Validated in a High-Altitude Environment:** The hardware was deployed and validated under the adverse climatic conditions of the Peruvian high-Andean tundra, demonstrating its robustness against low temperatures, high humidity, and significant UV exposure.

#### 7.6.2 Limitations

• **Long-Term Component Durability:** Although the system demonstrated consistent reliability during the intensive 30-day validation campaign and subsequent intermittent checks over a one-year period, a continuous multi-year degradation study has not been conducted. The long-term UV resistance of the 3D-printed PETG enclosures and the lifespan of the capacitive soil sensors under perpetual electrolysis remain variables that require extended longitudinal assessment.
• **Sensor Drift and Degradation:** The consumer-grade sensors are susceptible to physical degradation and measurement drift over time, particularly those exposed directly to soil moisture and UV radiation. A periodic recalibration and replacement strategy is necessary for maintaining data accuracy in long-term deployments.
• **Communication Bandwidth Constraints:** LoRa technology offers excellent range at the expense of a low data rate, which limits applications requiring high-frequency sampling (<1Hz). The nRF24L01 network operates in the 2.46 GHz ISM band, making it susceptible to interference if deployed near dense Wi-Fi networks.
• **Basic Anomaly Detection:** The current software relies on validation at the Main Base and simple error counters at the node level. It lacks advanced, real-time algorithms on the edge devices for sophisticated anomaly detection or sensor fault diagnosis.
• **Interference Characterization:** While basic functionality was verified, no systematic characterization of packet loss under intentional high-power interference or massive multi-node scaling (>5 nodes) was performed.

#### 7.6.3 Future work and scalability strategy

Based on the current validation, the following enhancements and scaling strategies are proposed for future iterations:

• **Multi-Greenhouse Scalability Plan:** To deploy PyLoGreen across multiple adjacent greenhouses, a channelization strategy will be implemented. This involves assigning unique LoRa addresses (configurable via the E32 UART command set) and separating RF channels (e.g., 905 MHz, 915 MHz, 925 MHz) for each Main Base to prevent packet collisions. Furthermore, local nRF24L01 subnets will be isolated using distinct 5-byte pipe addresses.
• **Migration to LoRaWAN:** For large-scale deployments where internet infrastructure is available, the hardware is compatible with LoRaWAN Class A operation. Future firmware updates will implement the LoRaWAN MAC layer stack to enable centralized management of hundreds of nodes via a Network Server (e.g., The Things Network), replacing the current P2P topology.
• **Transition to Industrial-Grade Sensors:** To improve long-term reliability and accuracy, future versions could integrate industrial-grade sensors that use robust communication protocols like **RS-485**, which offers superior noise immunity in electrically noisy agricultural environments.
• **Enhanced Local Area Network:** The 2.46 GHz local network could be upgraded to a mesh networking technology (e.g., Thread or Zigbee) to improve reliability and extend the range within larger greenhouse structures.
• **Hardware Resource Optimization:** A systematic analysis will be performed to optimize the use of the I/O and processing capabilities of the Raspberry Pi. Furthermore, a cost–benefit analysis will be conducted to evaluate migrating from general-purpose hardware to a more dedicated, application-specific integrated system to further reduce costs and power consumption.

### 7.7 Conclusion

The PyLoGreen project represents a successful and documented implementation of a low-cost agricultural monitoring and control system, specifically designed to operate under the demanding climatic conditions of the high-Andean region. Its main strength lies in the pragmatic balance between cost, functionality, and robustness, achieved through well-justified engineering decisions. The hybrid communication architecture proves to be a highly effective and economical solution, and the validation in a real-world environment provides significant credibility to the results and demonstrates the resilience of the design.

Although inherent limitations exist in a low-cost design, primarily related to the long-term durability of its components, these do not diminish the value of the project. Instead, they highlight the necessity for planned maintenance, a realistic factor in any field-deployed technology. In conclusion, PyLoGreen is not only a functional prototype but also a validated and replicable model that demonstrates how open-source technology can create accessible and effective solutions. The project fulfills its goal of democratizing access to precision agriculture, providing a valuable tool for optimizing crops in regions where commercial solutions are economically unfeasible.

## CRediT authorship contribution statement

**Renzo Victor Carpio Hallasi:** Writing – review & editing, Writing – original draft, Supervision, Software, Resources, Project administration, Methodology, Investigation. **Yusef Mamani Arqque:** Writing – review & editing, Writing – original draft, Software, Resources, Methodology, Investigation, Conceptualization. **Franco Alessandro Arenas Mamani:** Writing – review & editing, Visualization, Validation, Formal analysis, Data curation. **Alejandro Enrique Contreras Corzo:** Writing – review & editing, Writing – original draft, Validation, Resources, Formal analysis. **Pablo Lizardo Pari Pinto:** Writing – review & editing, Writing – original draft, Validation. **Erasmo Sulla Espinoza:** Writing – review & editing, Writing – original draft, Validation.

## Ethics statements

This work did not involve human subjects or animal experiments.

## Declaration of competing interest

The authors declare that they have no known competing financial interests or personal relationships that could have appeared to influence the work reported in this paper.

## Figures and Tables

**Fig. A.1:**
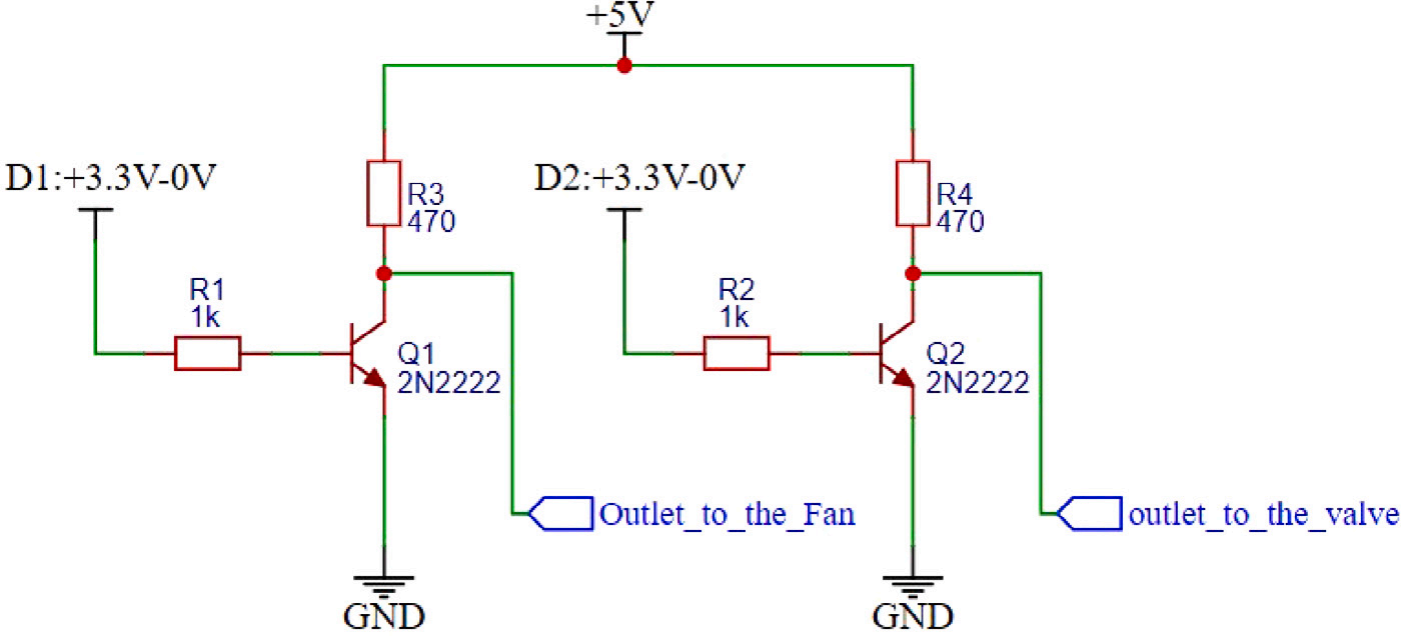
NPN transistor polarization.

**Fig. B.1:**
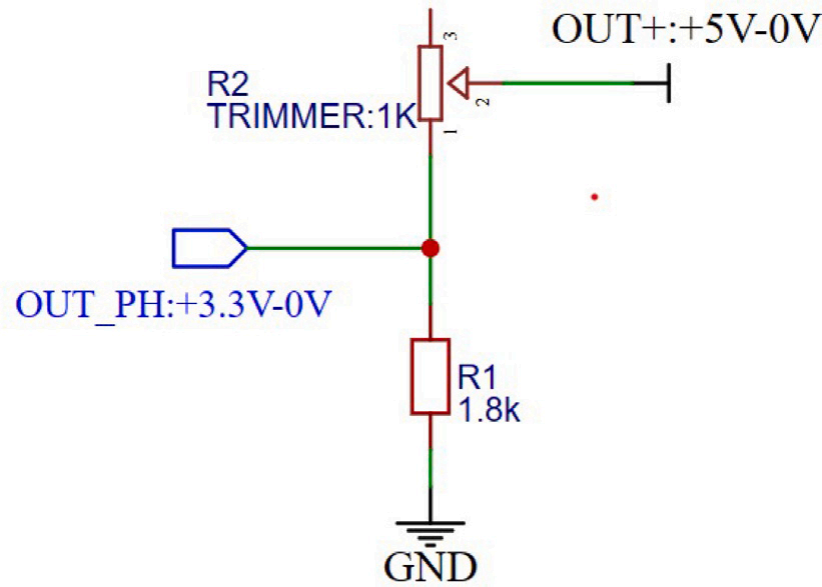
Voltage divider.

**Fig. C.1:**
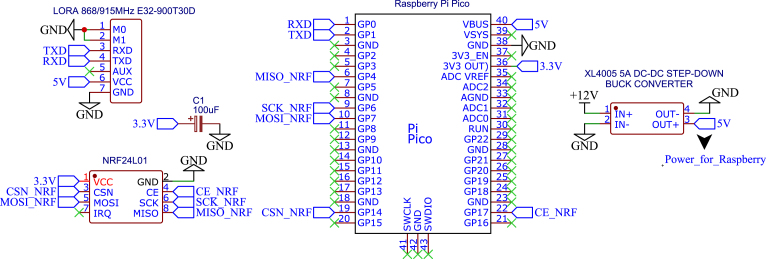
Schematic diagram Node 0.

**Fig. C.2:**
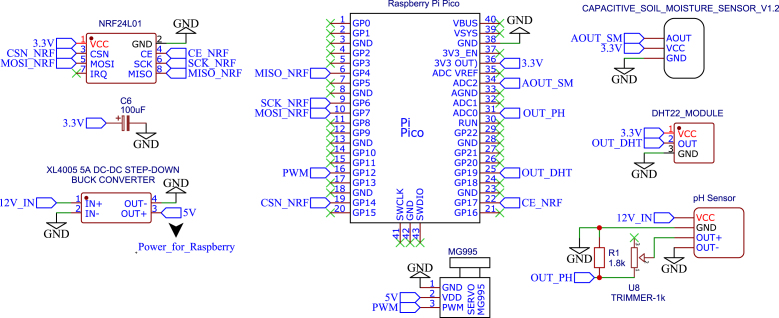
Schematic diagram Node 2.

**Fig. C.3:**
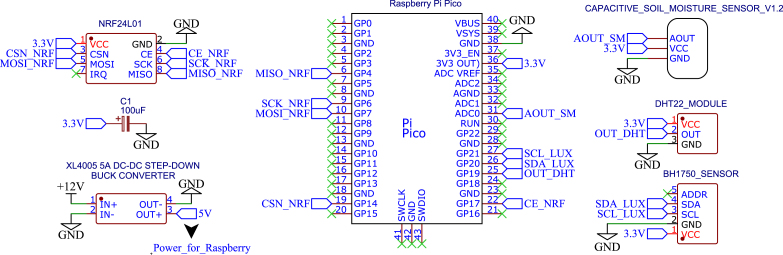
Schematic diagram Node 3.

**Fig. C.4:**
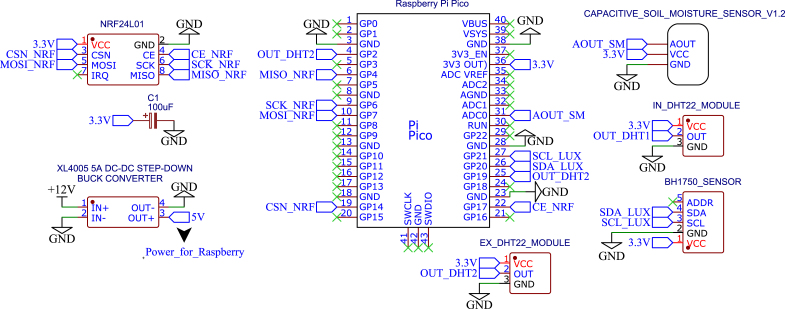
Schematic diagram Node 4.

**Fig. D.1:**
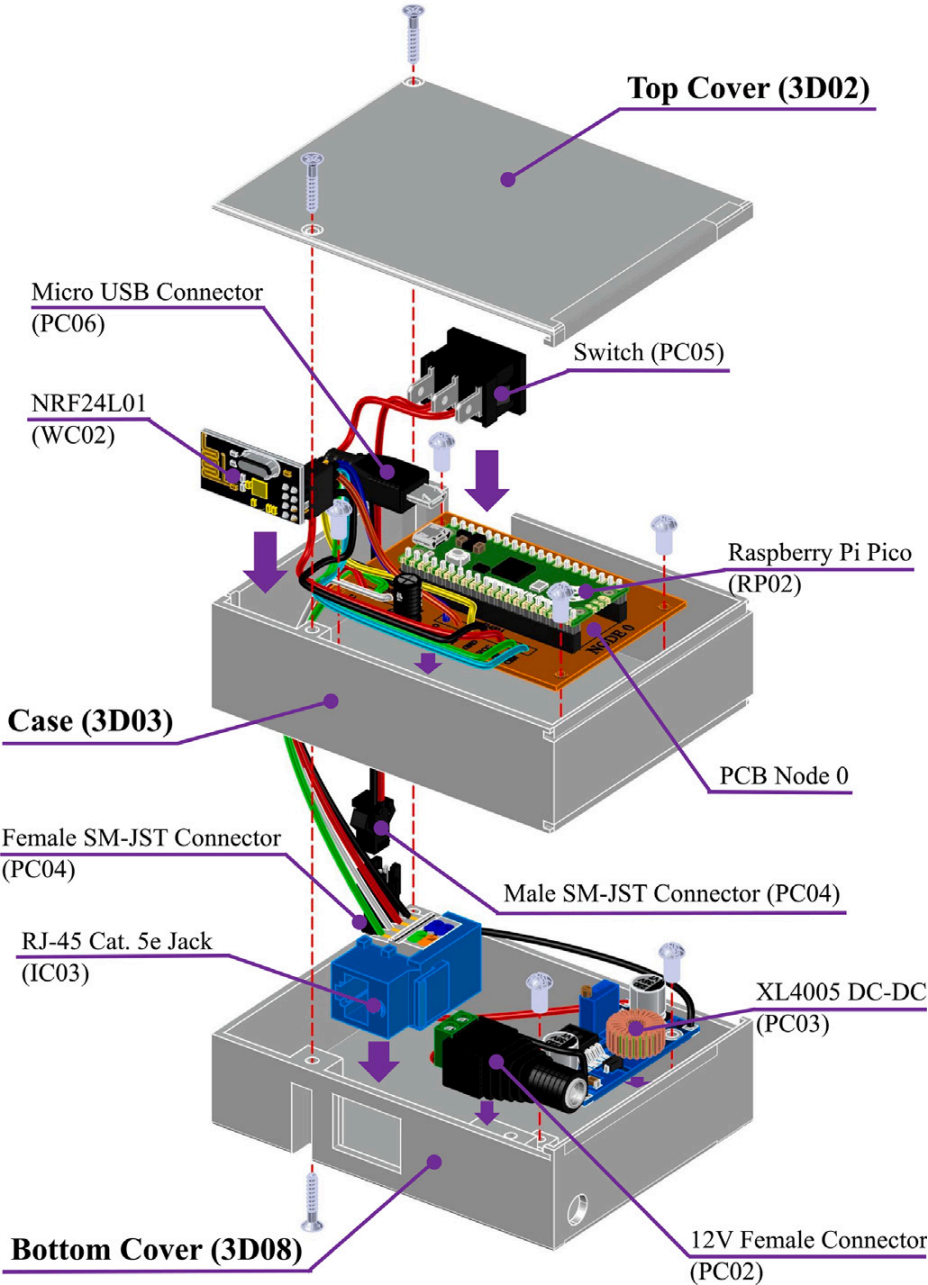
Node 0 Assembly.

**Fig. D.2:**
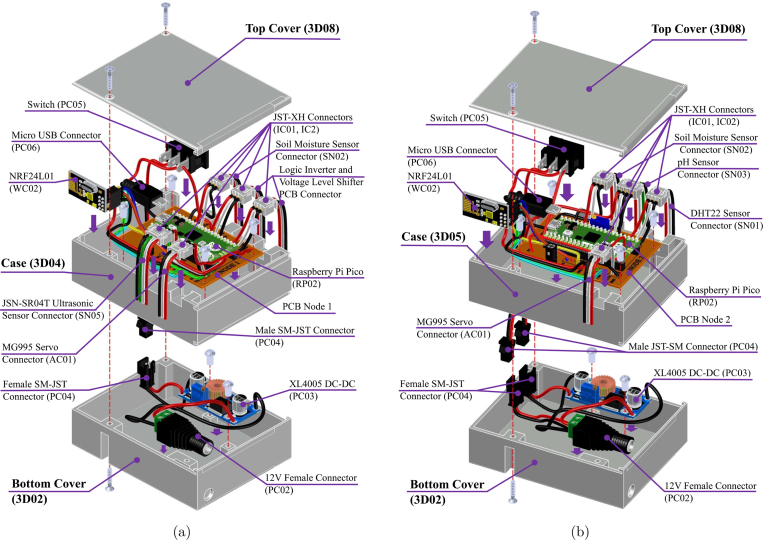
Assemblies of Nodes 1 and 2: (a) Node 1 Assembly, (b) Node 2 Assembly.

**Fig. D.3:**
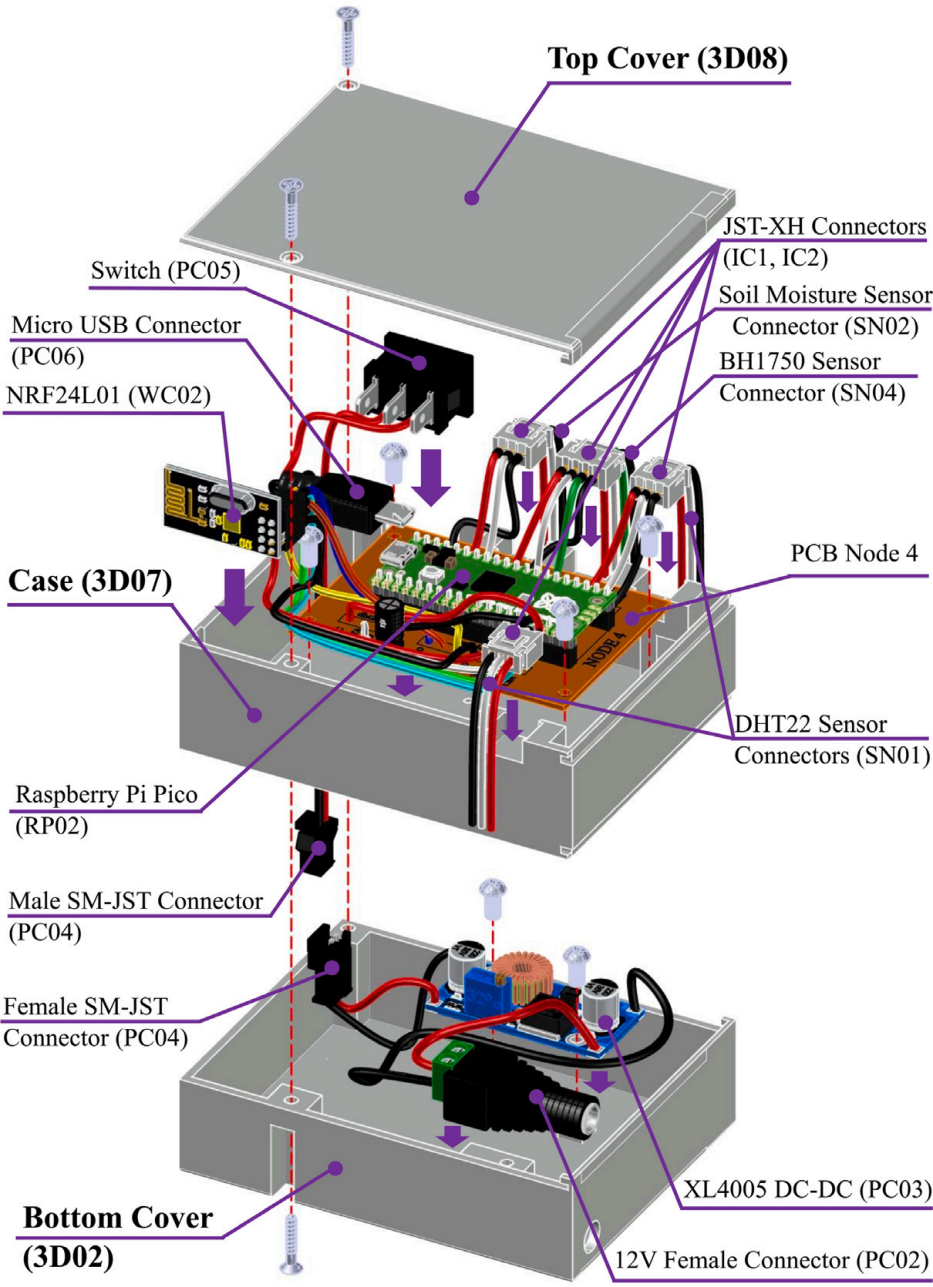
Node 4 Assembly.

**Fig. E.1:**
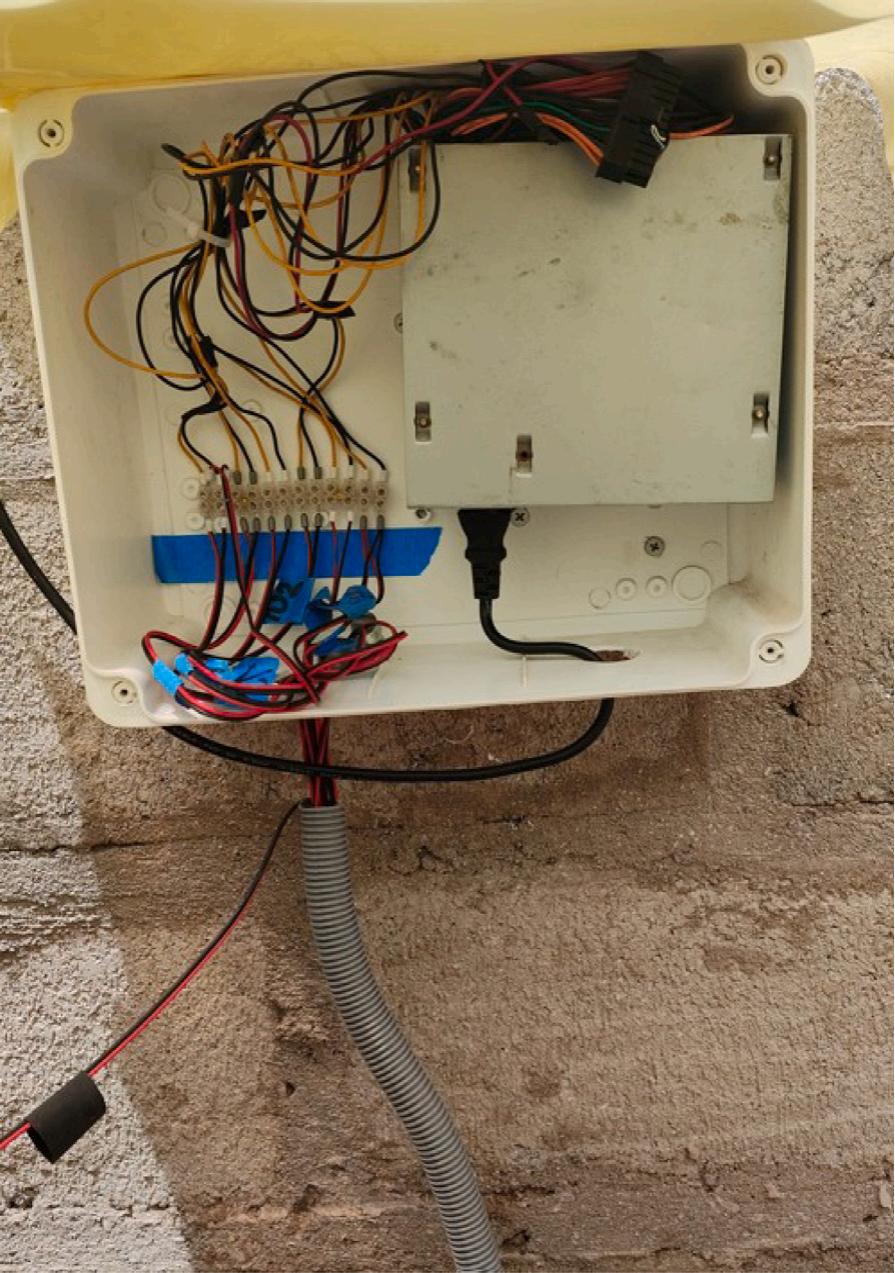
Project DC power supply.

**Fig. F.1.1:**
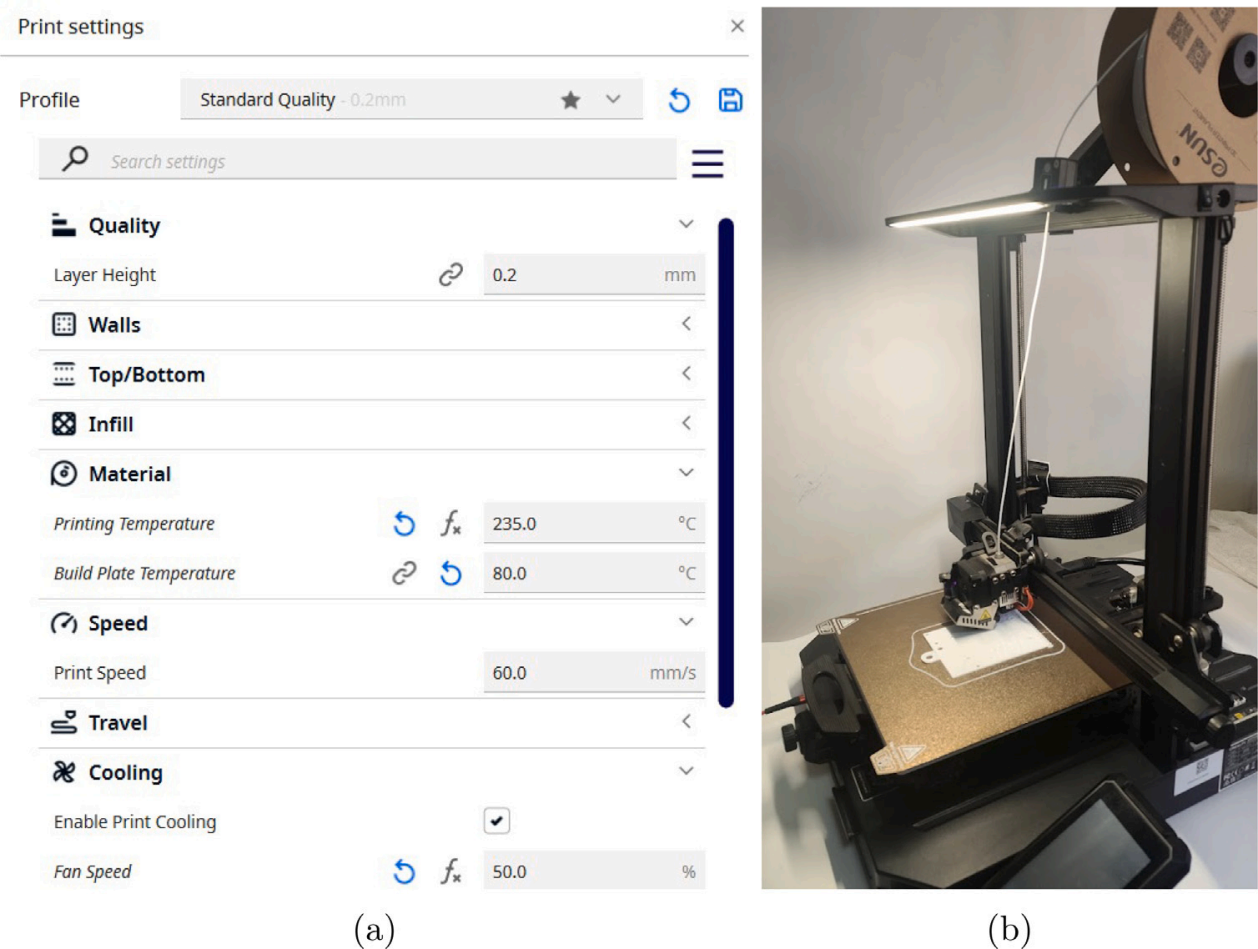
3D Printing: (a) Printing for PETG, (b) Printing Process.

**Table 5:** Required 3D-printed parts for electronic and sensor integration.

Component	Files and quantities
DHT22 sensor (SN01)	4× covers (3D14)
BH1750 sensor (SN04)	2× cases (3D10)
JSN-SR04T Ultrasonic Sensor (SN05)	1× case (3D16), 1× cover (3D17), 1× cover (3D18)
LoRa module (WC01)	2× cases (3D11), 2× covers (3D12)
LoRa antenna (WC05)	2× Antenna mounts (3D13)
RJ45 Connector (IC03)	1× support (3D15)

**Table 6:** Manufacturing files and dimensions for the custom PCB units.

PCB/Purpose	Qty.	Dimensions	Trace file	Silkscreen file
Node 0	1	50 × 70 mm	FL03	FL04
Node 1	1	50 × 70 mm	FL05	FL06
Node 2	1	50 × 70 mm	FL07	FL08
Node 3	1	50 × 70 mm	FL09	FL10
Node 4	1	50 × 70 mm	FL11	FL12
LoRa breakout board	2	20 × 22 mm	FL01	FL02
Logic inverter and voltage level shifter PCB	1	28 × 43 mm	FL13	FL14

**Fig. G.2.1.1:**
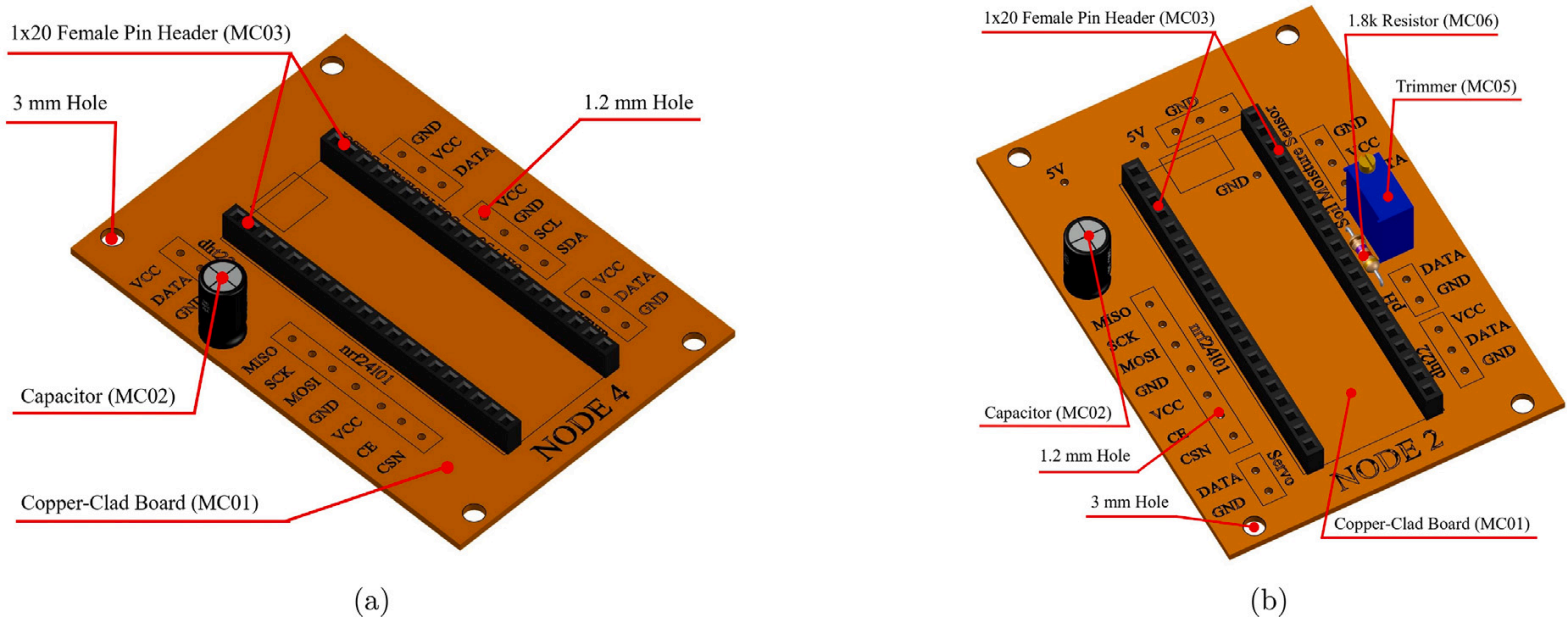
Base soldering: (a) Common layout for Node 4, (b) Additional sensor interface components for Node 2.

**Fig. G.2.2.1:**
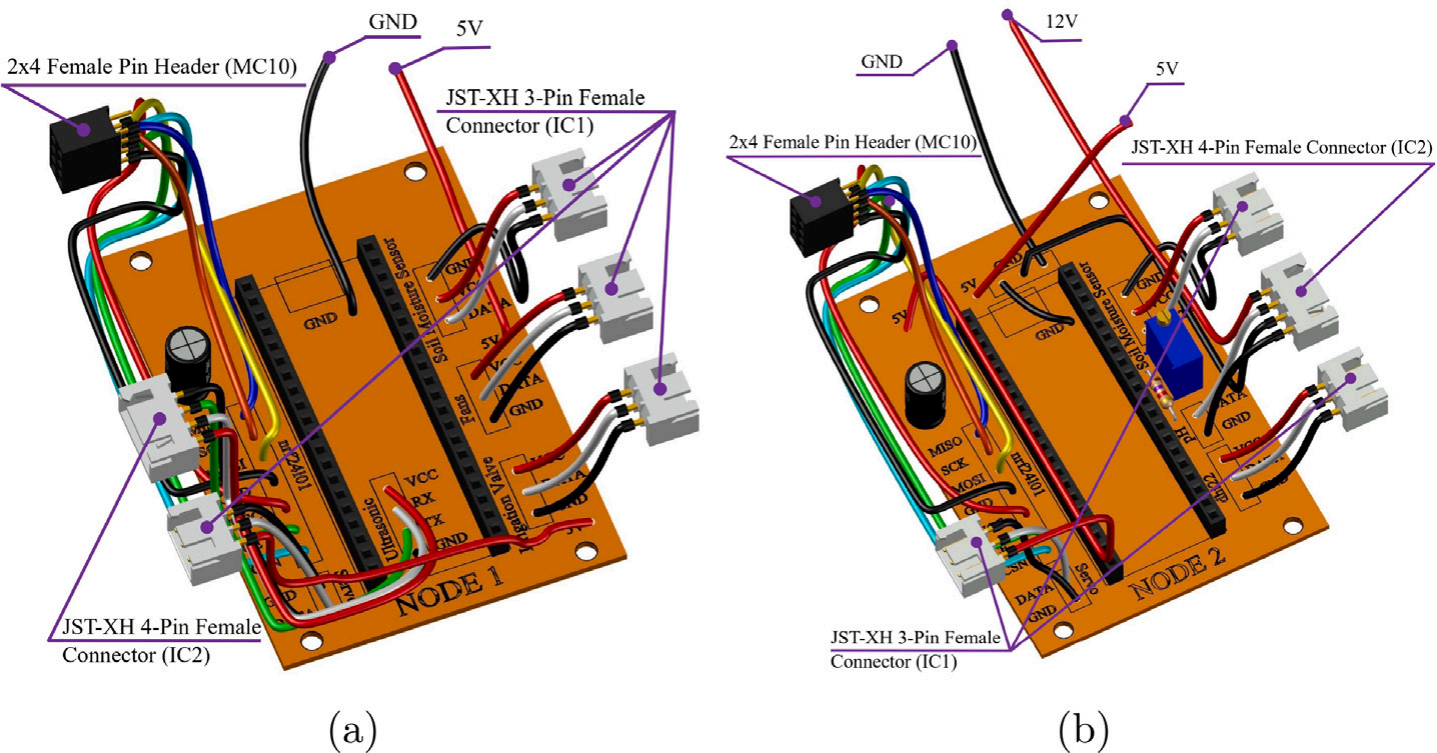
Soldered JST-XH connector mapping: (a) Node 1, (b) Node 2.

**Fig. G.3.1:**
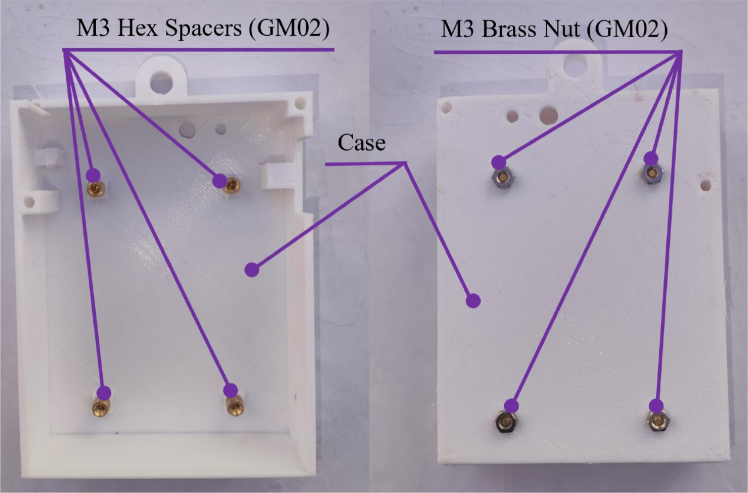
Installation of M3 hexagonal spacers in the enclosures.

**Fig. G.5.1:**
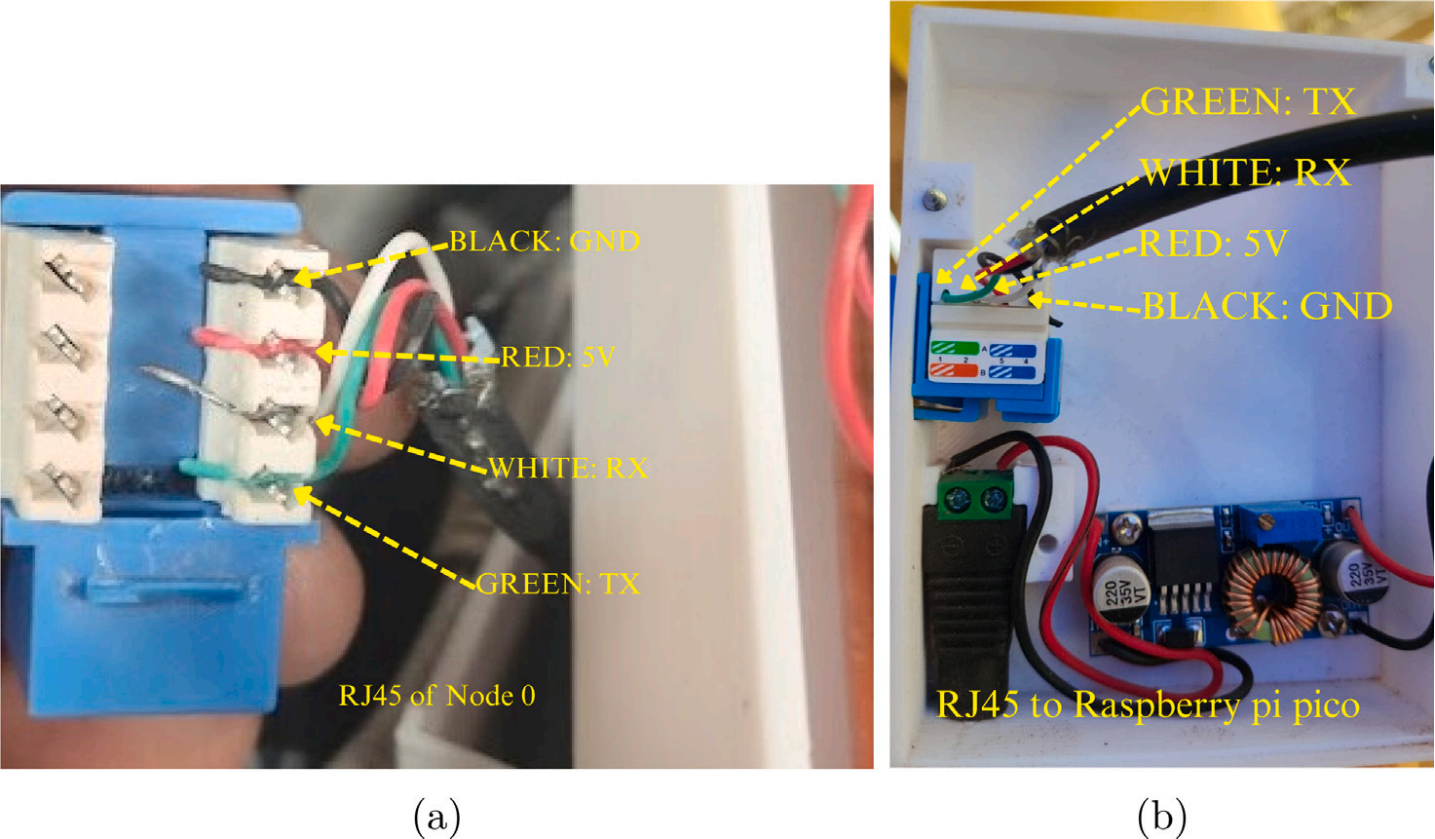
Detailed wiring color codes for the Gateway interface: (a) RJ45 Node 0 Wiring, (b) Internal Connection.

**Fig. G.5.1.1:**
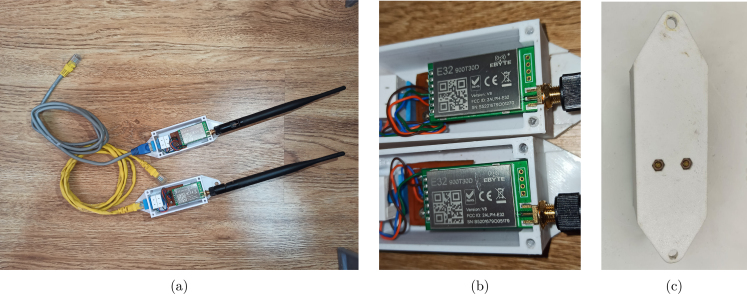
External LoRa Unit integration: (a) General assembly showing internal wiring and antennas, (b) Internal detail of the LoRa module and RJ-45 connection, (c) External view of the protective enclosure.

**Fig. G.5.2.1:**
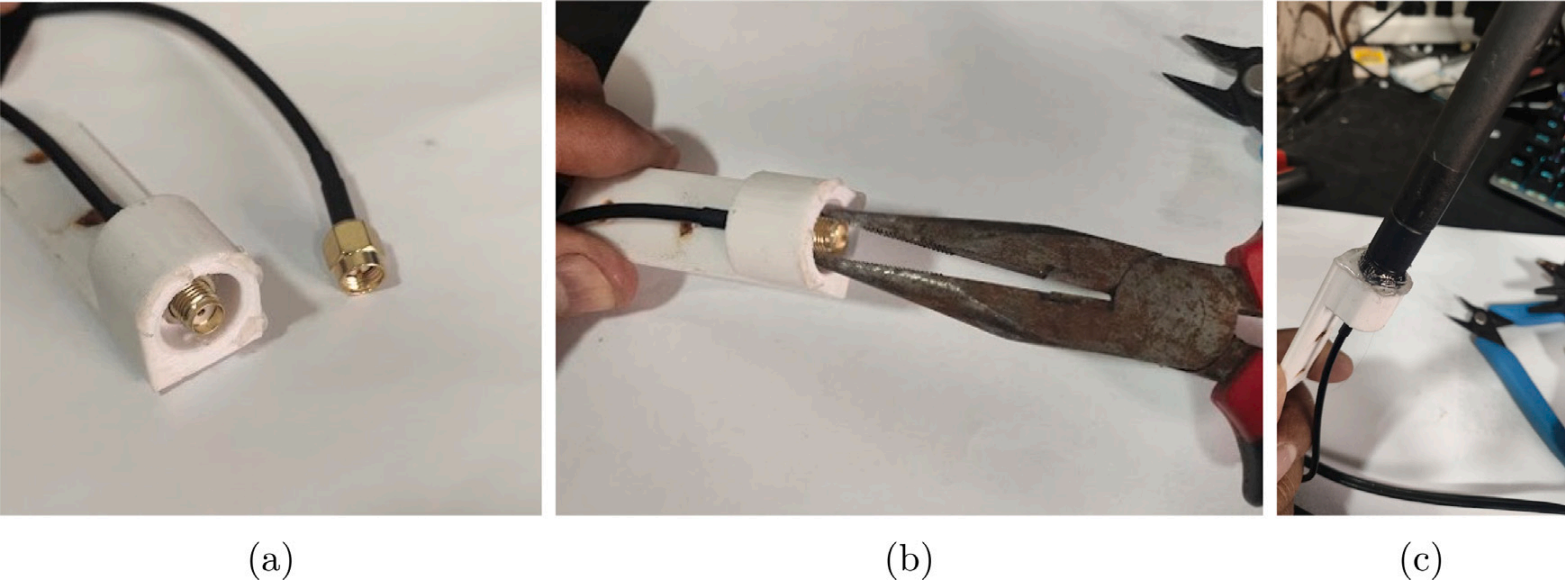
Antenna assembly: (a) Cable insertion, (b) Nut tightening, (c) Antenna placement and sealing.

**Fig. G.6.1:**
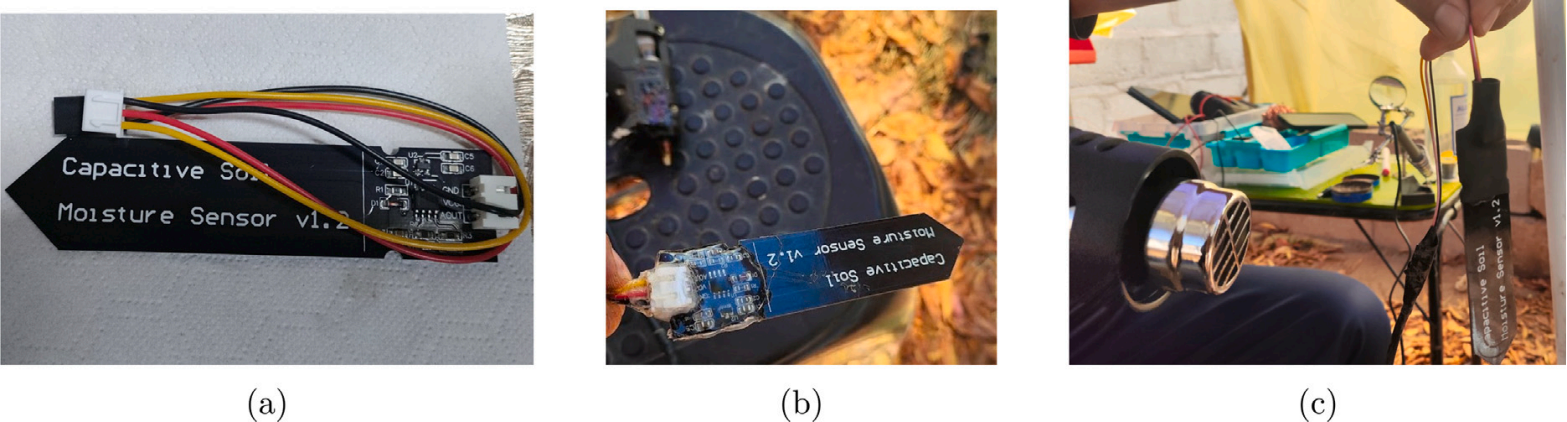
Waterproofing process for the capacitive soil moisture sensor: (a) Raw Sensor, (b) Silicone Coating, (c) Final Sealing.

**Fig. G.6.2:**
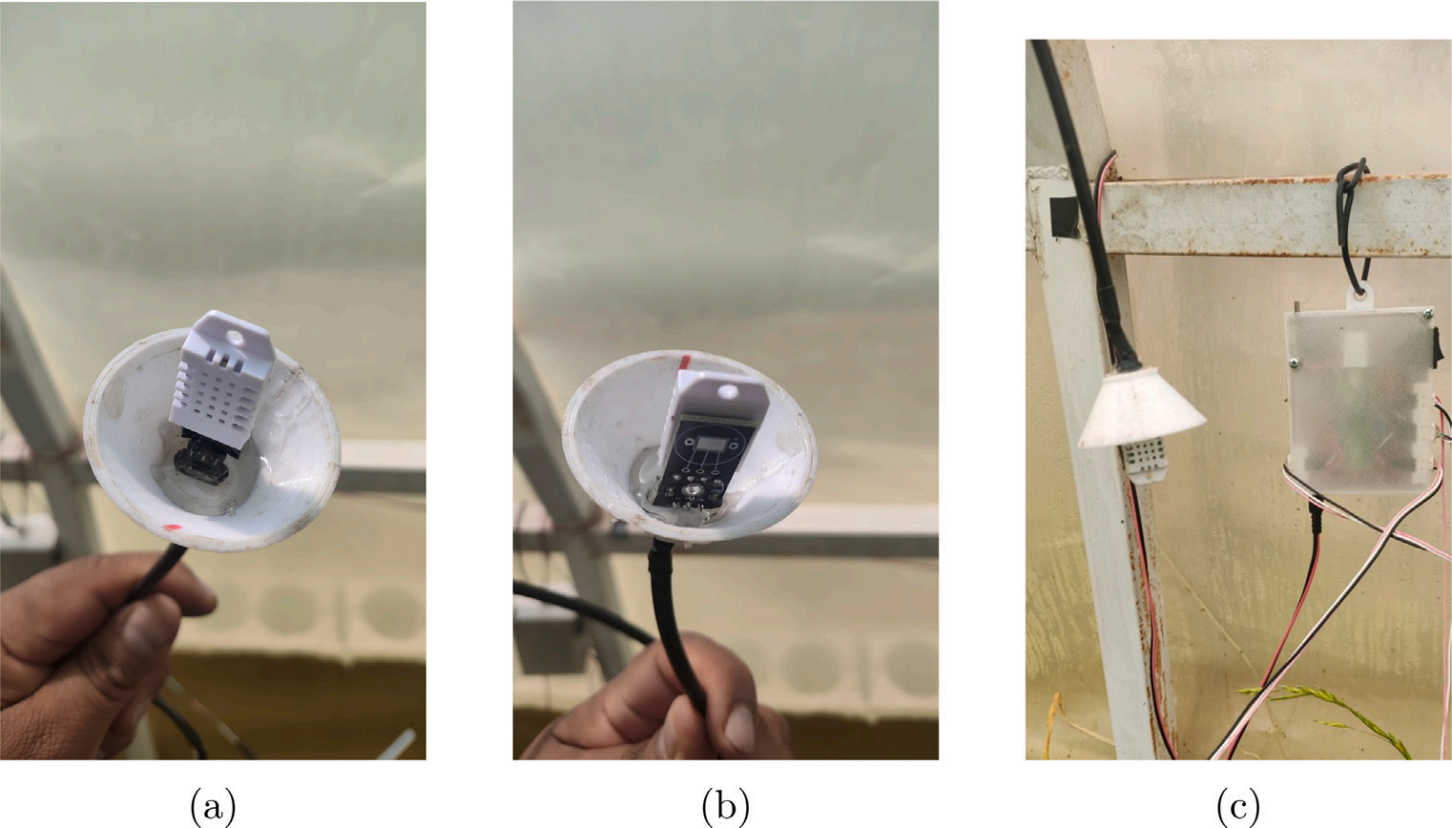
DHT22 sensor preparation: (a) Mounting, (b) Silicone, (c) Suspension.

**Fig. G.7.1:**
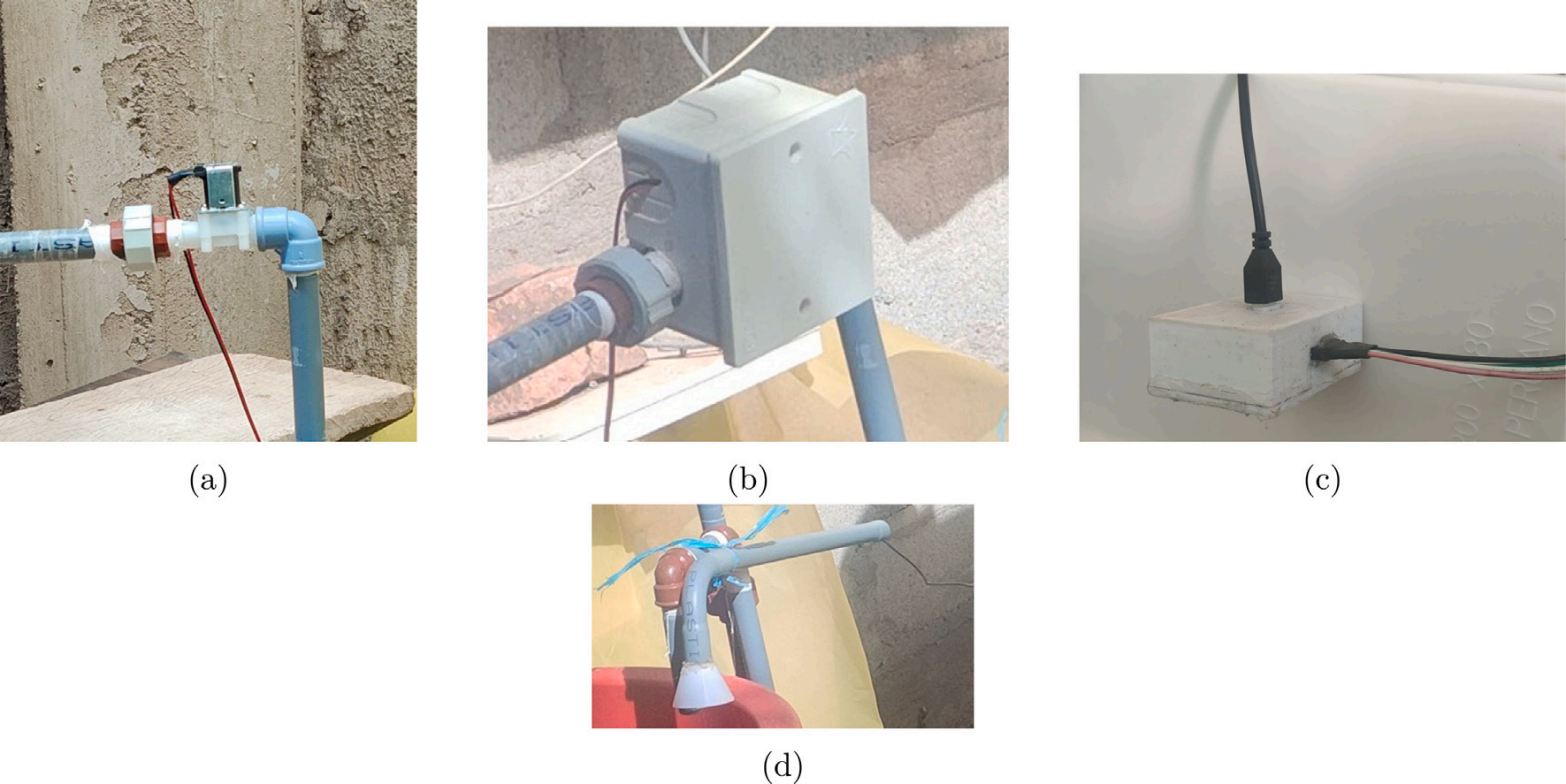
Mechanical integration of actuators and ultrasonic sensors: (a) Valve, (b) Valve Box, (c) Sensor Cover, (d) Joint.

**Table 7:** Software files required for each sensor node.

Node ID	Main script (rename to main.py)	Required libraries	
Node 0	main_node_0.py (PY02)	nrf24l01reg.py (PY07)	
Node 1	main_node_1.py (PY03)	nrf24l01reg.py (PY07)	
Node 2	main_node_2.py (PY04)	nrf24l01reg.py (PY07)	dht.py (PY08)
Node 3	main_node_3.py (PY05)	nrf24l01reg.py (PY07)	dht.py (PY08)
Node 4	main_node_4.py (PY06)	nrf24l01reg.py (PY07)	dht.py (PY08)

**Table 8:** Required modifications in main_base.py for replication.

Line(s)	Required action
20	UART Port: Change ‘/dev/serial0’ to the host port (e.g., ‘COM3’ on Windows).
77, 81	Credentials: Update the path to serviceAccountKey.json and the Database URL.
84–89	References: Match the database paths to your Firebase structure.
161	Logging: Set a valid local path for the backup text file.

**Fig. H.1:**
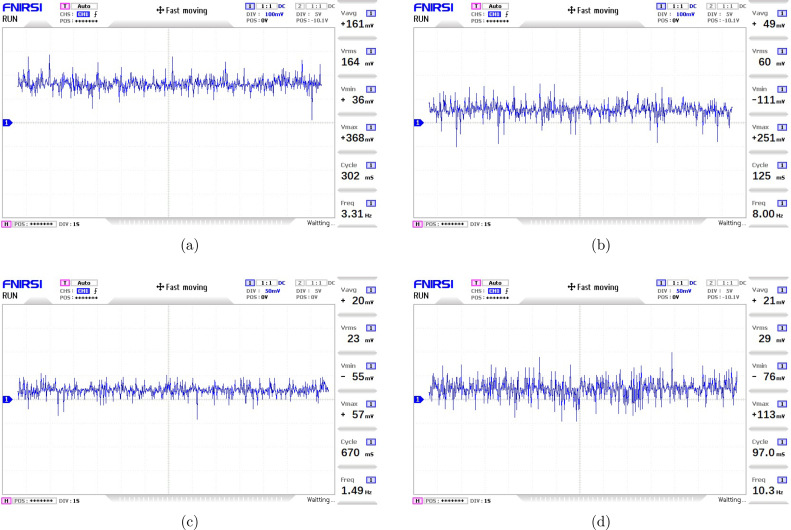
Measured current profiles for nodes 1-4. The baseline noise is characteristic of the step-down DC-DC converter operating at light loads: (a) Node 1, (b) Node 2, (c) Node 3, (d) Node 4.

## Appendix A Calculation of the NPN transistor biasing

To ensure the biasing of the transistor [23], in the circuit of Fig. A.1 the base current ($I_{B}$) is calculated using Eq. (A.1), ensuring that the device operates in the active region.

(A.1) $$I_{B}=\frac{V_{BB}-V_{BE}}{R_{B}}$$

Replacing the data:

$$I_{B}=\frac{3.3\;\text{V}-0.7\;\text{V}}{1\;\text{k}\Omega }$$

$$I_{B}=2.6\;\text{mA}$$

Since the transistor is biased (datasheet reference), the collector current is:

(A.2) $$I_{C}=\frac{V_{CC}-V_{CE}}{R_{C}}$$

$$I_{C}=\frac{5\;\text{V}-0.2\;\text{V}}{470\;\Omega }$$

$$I_{C}=10.2\;\mathrm{mA}$$

## Appendix B Calculation of pH and voltage divider

(B.1) $$V_{\text{OUT\_PH}}=V_{\text{OUT+}}\cdot \frac{R_{2}}{R_{1}+R_{2}}$$

Substituting the circuit values (with the trimmer $R_{2}$ tuned to 927 Ω to achieve the desired scaling):

(B.2) $$V_{\text{OUT\_PH}}=V_{\text{OUT+}}\cdot \frac{1800\;\Omega }{1800\;\Omega +927\;\Omega }$$

(B.3) $$V_{\text{OUT\_PH}}=0.66V_{\text{OUT+}}$$

Using the circuit shown in Fig. B.1, the voltage divider expression given in Eq. (B.1) was used to calculate the value of $V_{OUT\text{\_}PH}$. The result in Eq. (B.3) is a linear equation that allow us to find the pH, according with the requirements specified in the datasheet of the analog pH sensor [18], ensuring its correct operation and calibration thanks to the 1 kΩ trimmer.

(B.4) $$pH=\left(V_{\text{OUT\_PH}}/3.3\right)\times 6+3$$

For example, considering $V_{\text{OUT+}}=2.5\;V$, substituting this value into Eq. (B.3) yields $V_{\text{OUT\_PH}}=1.65\;V$. Then, inserting this result into Eq. (B.4) gives a corresponding pH value of approximately pH≈7.54.

## Appendix C Schematic diagrams of nodes

Schematic diagrams of the remaining nodes (see Fig. C.1, Fig. C.2, Fig. C.3, Fig. C.4):

## Appendix D Assembly for each node

The assembly and setup of each node are shown below. Although the sensors are not displayed, the connector from each sensor cable is visible and labeled accordingly.

Node 0: This node does not include sensors; it is responsible for processing data and performing the main intercommunication function (see Fig. D.1).

Nodes 1 and 2: These nodes include sensors and actuators, and are responsible for control and monitoring (see Fig. D.2).

Node 4: This node serves as a dedicated monitoring unit, responsible for collecting data from both inside and outside the greenhouse. In contrast, Node 3 also performs monitoring, but its function is limited to internal data collection exclusively (see Fig. D.3).

## Appendix E DC power supply

To power the entire system with direct current (DC), a 350 W computer ATX power supply was used, connected to the 220 V AC mains. The 12 V DC voltage was obtained from the yellow wires, using the black wires as ground (GND), as shown in Fig. E.1.

The power distribution was carried out through a terminal block, where the outputs were meticulously labeled for powering each node, as well as the solenoid valves and fans.

It is worth noting that this power configuration is flexible. Depending on availability and specific requirements, it can be replaced by other renewable energy sources, such as a photovoltaic system (solar panels with 12 V batteries) or even small-scale wind generators.

## Appendix F Detailed 3D printing protocol

This appendix provides the technical specifications and procedural steps required to manufacture the structural components of the PyLoGreen system using Fused Deposition Modeling (FDM).

### F.1 Material selection and printing parameters

For the fabrication of the structural components, white PETG filament (GM04) was selected. This material offers superior thermal resistance and mechanical durability compared to standard PLA. Furthermore, the white color was specifically chosen to maximize the reflection of solar radiation, providing passive thermal protection to the internal electronics against the high UV index of the deployment region. All parts were printed using a Creality Ender-3 S1 Pro 3D printer, as shown in Fig. F.1.1(b), and the UltiMaker Cura V.5.6.0 software.

The recommended printing settings, detailed in Fig. F.1.1(a), specify a 0.2 mm nozzle. The printing temperature should be set to 235 °C, the heated bed to 80 °C, and the print speed to 60 mm/s, keeping the layer fan at 50%.

### F.2 Recommended printing sequence

To optimize manufacturing time, it is recommended to follow the order listed below once the printer is properly leveled and calibrated.

#### F.2.1 Main node enclosures

1. Main Enclosures (in order): Print Case Node 1 (3D04), Case Rele (3D09), Case Node 2(3D05), Case Node 3 (3D06), Case Node 4(3D07) and Case Node 0 (3D03). *Note: The relay box lid is a 112 × 112 mm acrylic sheet and is not 3D-printed.*
2. Bottom Covers: Print 1 cover (3D01) and 4 covers (3D02).
3. Top Covers: Print 5 covers (3D08).

*Tip:* While the printer handles these long-duration prints, you may proceed with the PCB fabrication described in the subsequent appendix.

#### F.2.2 Enclosures and brackets for electronic peripherals

The secondary components required to secure the electronics and sensors are detailed in Table 5.

## Appendix G Detailed fabrication guide

This appendix provides the technical specifications and step-by-step procedures required to manufacture the custom PCBs and integrate the electromechanical subsystems of the PyLoGreen platform. **Safety and Environmental Warning:** This procedure involves the use of Ferric Chloride (III) (GM05), a corrosive chemical. Strict adherence to the following safety protocols is mandatory:

1. **Personal Protective Equipment (PPE):**
  • Safety goggles (ANSI Z87.1 rated) to prevent eye damage from splashes.
  • Nitrile or neoprene gloves (standard latex is NOT resistant to FeCl${}_{3}$).
  • Long-sleeved lab coat or apron to protect skin and clothing.
2. **Ventilation:** Work ONLY in a well-ventilated area or fume hood. The etching process can release corrosive vapors (HCl), especially if the solution is heated or agitated.
3. **Alternative for Inexperienced Users:** If access to proper safety equipment or chemical disposal facilities is limited, we strongly recommend ordering the PCBs from a professional fabrication service (e.g., JLCPCB, PCBWay) using the provided Gerber files. The cost is approximately $5-$15 for a batch of 5 boards.

### G.1 PCB fabrication via toner transfer

To fabricate the printed circuit boards (PCBs) for this project, the editable files JS01 through JS07 are provided. The economical toner transfer method will be used, and this process will use the PDF files (FL01 through FL14). The specific dimensions and file mapping for each unit are detailed in Table 6

#### G.1.1 Step-by-step fabrication procedure

**Safety Warning:** Chemical etching involving Ferric Chloride (III) (GM05) must be performed in a well-ventilated area using protective gloves and eyewear. Dispose of chemical waste according to local environmental regulations.

1. **Printing:** Print the trace and silkscreen files on high-glossy paper at 100% scale (actual size) using a laser printer. High toner density is required for effective masking.
2. **Board Preparation:** Cut the copper-clad phenolic boards (MC01) to the dimensions specified in Table 6. Clean the copper surface thoroughly with isopropyl alcohol to remove oxidation and grease.
3. **Trace Transfer:** Align the printed traces with the copper side of the board. Apply uniform heat using an iron (approx. 180 °C) for 5–10 min. Submerge the hot board in cold water until the paper softens and can be peeled off, leaving the toner mask on the copper.
4. **Chemical Etching:** Immerse the boards in ferric chloride (III) (GM05) until all exposed copper is dissolved (approximately 15 min or more). Rinse the boards thoroughly with plenty of water and dry them.
5. **Silkscreen Transfer:** Repeat step 3, but this time using the silkscreen prints on the non-copper side of the board (component side).
6. **Drilling:** Finally, drill the holes in the boards as shown in Fig. G.2.1.1. Use a 1.2 mm drill bit for components and a 3 mm drill bit for mounting screws.

### G.2 PCB soldering guide

This section details the electronic population of the custom-designed PCBs for the five nodes and auxiliary control modules. Precision in soldering is required to ensure signal integrity across the LoRa and sensor interfaces.

#### G.2.1 Base components and MCU preparation (Nodes 0-4)

Before node-specific configuration, a common set of base components must be prepared and installed on all five PCBs:

• **MCU Sockets:** Cut the 1 × 40 female headers (MC01) into 1 × 20 segments. Solder these segments to the PCB to serve as sockets for the Raspberry Pi Pico (RP02). Correspondingly, solder 1 × 20 male headers (MC04) to each side of the MCU.
• **Decoupling Capacitors:** Solder the 100 μF capacitor (MC02) onto the designated pads of each node PCB to filter power noise (Fig. G.2.1.1a).
• **Radio Interface:** Solder 2 × 4 female headers (MC10) for the nRF24L01 module using 7–10 cm flexible wires. This allows for optimal positioning within the enclosure to minimize interference.
• **Node 2 Additional Hardware:** Solder the 4.7 kΩ resistor (MC06) and the 10  kΩ calibration trimmer (MC05) specifically on the Node 2 PCB (Fig. G.2.1.1b).

#### G.2.2 Node-specific connectors and internal wiring

While the nodes share a common PCB architecture, each unit requires a specific configuration of JST-XH connectors to be soldered according to its dedicated monitoring or control function (Fig. G.2.2.1).

• **Node 0:** Solder a 10–15 cm 4-wire interface cable (IC04) directly to the PCB for communication with the RJ-45 jack (IC03).
• **Node 1:** Solder four 3-pin JST-XH connectors (IC01), one 4-pin JST-XH connector (IC02), and the main power leads (red/black).
• **Node 2:** Solder three 3-pin and one 4-pin JST-XH connectors. **Important:** In addition to the standard 5 V and GND power wires, solder a separate loose wire to the 12 V pin for the final connection to the power jack (PC04), as depicted in Fig. G.2.2.1b.
• **Node 3:** Solder two 3-pin and one 4-pin JST-XH connectors.
• **Node 4:** Solder three 3-pin and one 4-pin JST-XH connectors, as shown in Fig. 9.

### G.3 Enclosure & power assembly details

**Power Wiring Specifics:**

• **Node 1:** Solder the loose 5 V wire to the switch terminal and splice the GND wire to the main power supply ground.
• **Node 2:** Install an SM-JST connector for the pH sensor (SN03). Solder the 12 V and GND wires to the connector pins as indicated in the schematics.

**Mechanical Mounting:** Screw the M3 hexagonal spacers (GM02) into the 3D printed mounting holes. The PCBs are secured onto these spacers to prevent short circuits with the case bottom (see Fig. G.3.1).

### G.4 Actuator control assembly guide

• **Mounting:** Secure the Logic Inverter PCB with two bolts (GM03). Install the two-channel relay (AC04) using four bolts, and the single-channel relay (AC05) with one bolt.
• **Power Wiring:** Install two JST-XH connectors (IC01). Solder the 5 V (red) and GND (black) wires to distribute power to both the relay modules and the Logic Inverter PCB.
• **Signal Wiring:** Connect the data wires for the valve (AC02) and fans (AC03) first to the Logic Inverter inputs, then route the outputs to the respective relay triggers.
• **Final Closing:** Place the acrylic cover (GM06) and fasten it with four screws (GM01) as shown in Fig. 13(b).

### G.5 Main base wiring guide

Custom UTP cables are required to link the Main Base to Node 0. The wiring follows the T-568B connector standard but uses a custom pin definition:

**Raspberry Pi 4B Connection (Base):**

• **Power:** Blue-White wire → 5 V (Pin 4); Blue wire → GND (Pin 6).
• **Data:** Orange-White wire → TX (Pin 8); Orange wire → RX (Pin 10).

**Node 0 Connection (Gateway):** The flexible cable (IC04) inside Node 0 maps the UTP signals to the Raspberry Pi Pico (RP02):

• **5 V:** Red wire (Blue-White from UTP).
• **GND:** Black wire (Blue from UTP).
• **TX (GP1):** Green wire (Orange-White from UTP).
• **RX (GP0):** White wire (Orange from UTP) (see Fig. G.5.1).

#### G.5.1 External LoRa module enclosure

To maximize RF range and ensure line-of-sight connectivity, the LoRa transceivers are housed in dedicated external enclosures (Fig. 15), separate from the main processing units. This modular design isolates the RF components from the digital noise of the Raspberry Pi and allows for high-elevation mounting.

The enclosure integrates a custom adaptor PCB secured via hexagonal spacers to ensure mechanical stability. Connectivity is established through an RJ-45 interface, which carries power and SPI data from the main node. The assembly is designed to be weather-resistant, with the LoRa module positioned near the top cover to facilitate the connection to the external antenna (see Fig. G.5.1.1).

#### G.5.2 External LoRa module enclosure

**LoRa 915 MHz Antenna Connections:** The antenna assembly is the same for the Base and for Node 0. The SMA coaxial cable WC03 (for Node 0) and the SMA coaxial cable WC04 (for the Base) are installed with the printed part 3D13, as shown in Fig. G.5.2.1(a) (female end of the SMA coaxial cable). Next, the nut must be tightened with a long plier, as shown in Fig. G.5.2.1(b). Finally, the antenna (WC05) is screwed in and sealed with a small amount of silicone, as shown in Fig. G.5.2.1(c), to protect the antenna.

### G.6 Sensor waterproofing and assembly

• **Soil and Atmospheric Sensors:** The capacitive soil moisture sensors (SN02) and DHT22 modules (SN01) underwent a conformal coating process using silicone sealant and heat-shrink tubing to hermetically seal the electronic components while keeping the sensing elements exposed.
• **Actuation and Distance Monitoring:** The irrigation control valves (AC02) are protected within IP65-rated junction boxes (GM08). Similarly, the ultrasonic water level sensor (SN05) features a custom 3D-printed housing (3D16) to shield the circuitry from condensation in the water tank.
• **Light Sensors:** BH1750 modules (SN04) were mounted on angled 3D-printed brackets (3D10) to optimize incident light capture and prevent water accumulation on the sensor surface.

**Capacitive Soil Moisture Sensor (SN02):** To prevent oxidation of the sensor electronics in wet soil:

1. Reuse the 3-pin male connector (Fig. G.6.1(a)).
2. Solder a 1.5–2 m flexible cable (IC04): Red (VCC), Black (GND), Yellow/White (Data).
3. Apply a generous layer of silicone sealant over the top electronics (Fig. G.6.1(b)).
4. Seal the entire upper assembly with heat-shrink tubing (Fig. G.6.1(c)) for complete watertightness.

**DHT22 Temperature/Humidity Sensor (SN01):**

• Mount the sensor into the 3D printed holder 3D14.
• Apply silicone to the cable junction (Fig. G.6.2(b)) to prevent moisture from traveling up the wire.
• Suspend the assembly inside the greenhouse using a 3-pin connector (IC01) as shown in Fig. G.6.2(c).

### G.7 Actuators and auxiliary sensors

**Irrigation and Ventilation:** The system controls servo-driven gates (AC01) and solenoid valves.

• **Valves:** The drip irrigation valve (AC02, Fig. G.7.1(a)) is housed in a junction box (GM08, Fig. G.7.1(b)) to protect electrical contacts from rain/sprinklers.
• **Ultrasonic Sensor:** The JSN-SR04T (SN05) uses a custom 3D printed cover (3D16, 3D17) and an elbow joint (3D18) sealed with silicone (Fig. G.7.1(d)) to monitor water tank levels without corrosion.

**BH1750 Light Sensors:** Sensors are mounted on brackets (3D10) using 3  mm screws. Shielded cables (IC04) are used: 1.5 m for indoor and 3 m for outdoor units to prevent interference.

### G.8 Software customization guide

**Firmware File Mapping:** Ensure the following files are uploaded to the root directory of the Raspberry Pi Pico.

To replicate the system, specific parameters in the source code must be adapted to the host environment. The following table identifies the exact lines in main_base.py requiring user modification (see Table 8).

### G.9 Final commissioning checklist

Before permanent installation, verify the following:

1. **Voltage Regulation:** Confirm that all step-down DC-DC modules (PC03) output exactly 5.0 V. Over-voltage will destroy the Raspberry Pi Pico.
2. **Address Verification:** Perform a bench test via USB to confirm that each node responds to its specific ID (0-4) before sealing the enclosures.
3. **LoRa Signal:** Verify the RSSI levels between Node 0 and the Main Base. Ensure the antennas are vertically polarized and have a clear line of sight.

## Appendix H Supplementary power profiles

This appendix presents the current consumption oscillograms for the distributed sensor nodes (see Fig. H.1).

## Appendix I Glossary

This glossary provides definitions for the key technical and contextual terms used throughout this article.

• *3D printing:* Additive manufacturing process for producing custom plastic enclosures and mounts.
• *BH1750:* Digital light intensity sensor that communicates via I2C protocol.
• *Biasing:* Adjustment of transistor voltages to ensure correct switching or amplification.
• *Capacitive soil moisture sensor:* Analog sensor that measures soil water content based on dielectric properties.
• *Control signal (CS):* Encoded integer representing the control mode, thresholds, and actuator states of the system.
• *DC-DC Step-Down Converter (XL4005):* Voltage regulator that converts 12 V DC to 5 V DC for powering nodes.
• *DHT22:* Digital temperature and humidity sensor with 1-Wire communication protocol.
• *Etching:* Chemical process used to remove copper from a PCB during fabrication.
• *Firebase:* Cloud platform used for real-time data storage and visualization.
• *Greenhouse:* Covered structure that creates a protective microclimate for crop cultivation in high-altitude environments.
• *High-Andean Tundra:* An ecological region found in the high elevations of the Andes mountains, located at 3824 m above sea level (m.a.s.l.).
• *Hysteresis control:* Control strategy that uses two thresholds (lower/upper) to avoid rapid switching and ensure stable actuation (e.g., irrigation valve activation and deactivation).
• *I2C:* Serial communication protocol used by digital sensors such as BH1750.
• *ISM band:* Industrial, Scientific and Medical radio bands allocated for license-free operation under national regulations.
• *ISM:* Industrial, Scientific, and Medical.
• *JSN-SR04T:* Waterproof ultrasonic sensor used for non-contact distance or water level measurement.
• *LoRa:* Acronym for Long Range. A patented low-power wide-area network (LPWAN) communication protocol that utilizes Chirp Spread Spectrum modulation.
• *Main Base:* Central processing unit that coordinates sensor nodes and communicates with the cloud.
• *Microclimate:* Local environmental condition within the greenhouse that affects plant growth.
• *MicroPython:* Lightweight implementation of the Python language for microcontrollers.
• *Node:* Independent data acquisition module that measures environmental variables and transmits them wirelessly.
• *nRF24L01:* A widely used, compact 2.46 GHz radio transceiver module.
• *Payload:* Data content of a communication packet.
• *PCB (Printed Circuit Board):* Board that mechanically supports and electrically connects electronic components.
• *PETG:* Acronym for Polyethylene Terephthalate Glycol-modified. A thermoplastic polyester widely used in additive manufacturing (3D printing).
• *pH level:* Measure of soil acidity or alkalinity.
• *PNAF:* National Frequency Allocation Plan; defines permitted frequency allocations and constraints for radio operation in Peru.
• *PRR (Packet Reception Ratio):* Percentage of successfully received packets over a communication link during a test window.
• *PyLoGreen:* Low-cost open-source agricultural monitoring system based on Raspberry Pi and LoRa communication.
• *Python:* High-level programming language used for Main Base control and cloud communication.
• *Raspberry Pi 4 Model B (RPi 4B):* A high-performance Single-Board Computer (SBC) serving as the central hub or Main Base of the system.
• *Raspberry Pi Pico:* A low-cost, high-performance microcontroller board based on the RP2040 chip.
• *RMI (Relative Moisture Index):* Relative soil-moisture indicator obtained from a two-point calibration (dry reference and saturated reference); it is not an absolute volumetric water content measurement.
• *RS-PH-TR-1:* Analog soil pH transmitter operating from 3 to 9 pH units.
• *Silkscreen:* Printed labeling layer on the top of a PCB.
• *Soil moisture:* Amount of water present in the soil, expressed as a percentage.
• *Solenoid valve:* Electrically controlled valve for water irrigation management.
• *SPI:* Serial Peripheral Interface protocol used by transceivers like nRF24L01.
• *STL file:* Standard 3D model format used for 3D printing.
• *UART:* Universal Asynchronous Receiver-Transmitter communication protocol used between LoRa modules and Raspberry Pi.
• *Watchdog:* Software mechanism that resets a node after consecutive communication or reading failures.
